# Restoration of endogenous electric fields with a glucose-powered symbiotic bioabsorbable bandage for diabetic wound healing

**DOI:** 10.1126/sciadv.aed9445

**Published:** 2026-06-10

**Authors:** Lingling Xu, Engui Wang, Ming Ying, Lin Luo, Yongfang Ren, Chang Zhu, Jiaxuan Li, Tian Le, Hongqing Feng, Xia Wang, Chunying Chen, Zhou Li, Han Ouyang

**Affiliations:** ^1^ School of Nanoscience and Engineering, School of Chemical Sciences, University of Chinese Academy of Sciences, Beijing 100049, China.; ^2^ Vita Tech Innovation Center, Tsinghua Changgung Hospital, School of Clinical Medicine, Tsinghua University, Beijing 100084, China.; ^3^ New Cornerstone Science Laboratory, CAS Key Laboratory for Biomedical Effects of Nanomaterials and Nanosafety and CAS Center for Excellence in Nanoscience, National Center for Nanoscience and Technology, Beijing 100190, China.; ^4^ College of Materials Science and Opto-Electronic Technology, University of Chinese Academy of Sciences, Beijing 100049, China.; ^5^ School of Biomedical Engineering, Tsinghua Medicine, Tsinghua University, Beijing 100084, China.; ^6^ Beijing Institute of Nanoenergy and Nanosystems, Chinese Academy of Sciences, Beijing 101400, China.; ^7^ The Key Laboratory of Land Resources Evaluation and Monitoring in Southwest China, College of Geography and Resources, Sichuan Normal University, Chengdu 610066, China.; ^8^ Suzhou Institute of Nano-Tech and Nano-Bionics, Chinese Academy of Sciences, Suzhou 215123, China.

## Abstract

Endogenous electric fields (EFs) are essential for tissue regeneration but are diminished under hyperglycemia conditions, thereby impeding diabetic wound healing. Here, we report a biodegradable, glucose-powered electronic fabric bandage (GEB) that restores wound-edge electrical fields and enables closed-loop wound healing. To avoid compromising clinical applicability, we integrated all components into a soft, lightweight, and breathable bandage design to replace the traditional bulky electrical stimulator design. We also show the universal glucose-powered electricity generation and therapeutic functions of the electronic bandage across species and organs in diabetic wound models. In diabetic mouse wounds, porcine skin defects, and intestinal injury, the bandage uses endogenous glucose for power generation, thereby reducing local glucose levels and restoring the endogenous EF that guided cell migration, reprogrammed macrophage polarization, and promoted angiogenesis, to accelerate wound healing. These findings should establish an “endogenous glucose-powered symbiotic bioelectronics” paradigm for next-generation bioelectronic medicine.

## Teaser

A soft, breathable electronic bandage can be powered by endogenous energy substances (glucose) to repair diabetic wounds.

## INTRODUCTION

Nearly 800 million patients suffering from diabetes and its complications is a rising global threat (*1*–*4*). Beyond systemic metabolic dysregulation, diabetes severely impairs tissue repair capacity, with chronic, nonhealing wounds—particularly diabetic foot ulcers—posing one of the most serious clinical complications (*5*, *6*). In normal skin, endogenous electric fields (EFs) serve as key bioelectrical cues guiding wound healing: The negatively charged tissue surface generates a local potential gradient upon barrier disruption, driving ion currents that coordinate immune activation, cell migration, and tissue regeneration (*7*). In diabetic wounds, persistent hyperglycemia, oxidative stress, and inflammation disrupt ion transport and reduce EF potential, resulting in pro-inflammatory macrophage dominance, impaired cell proliferation and migration, and delayed tissue repair (*8*–*10*).

External electrical stimulation (ES) has emerged as an effective strategy to compensate for impaired EFs and accelerate healing (*11*–*13*). ES has been shown to reduce bacterial colonization and biofilm formation, restore normal wound closure in vivo, and enhance local tissue perfusion. It can also modulate immune cell activity, promoting a shift from pro-inflammatory to proregenerative phenotypes, and accelerate keratinocyte migration through a process known as electrotaxis. In addition, ES can stimulate angiogenesis and fibroblast proliferation, further supporting tissue repair and remodeling (*14*–*17*). However, conventional ES devices typically rely on bulky power sources, wired connections, and specialized clinical operation, which limit their portability, adaptability to dynamic movement, and continuous use in nonclinical settings, thereby restricting their broader application in routine wound management (*12*, *18*).

In response to these barriers, electrically active dressings have increasingly shifted from externally powered systems to wirelessly powered (*19*–*21*) or self-powered (*22*–*25*) designs to deliver continuous output and long-term therapy with minimal user burden. Glucose biofuel cell patches are particularly attractive because they harvest glucose from tissue fluid and wound exudate to provide sustained energy for prohealing ES. Recent studies have fabricated glucose biofuel cell patches by incorporating highly active catalytic systems or integrating microneedle architectures, thereby demonstrating the efficacy and safety of glucose-driven ES for wound repair (*26*, *27*). In parallel, to tackle the interface and materials bottlenecks that broadly constrain bioelectronic patches in practical use, substantial efforts have been devoted to improving soft conformal contact and wear comfort (*28*, *29*) and developing biodegradable constructs to broaden clinical applicability while minimizing secondary intervention such as device retrieval (*30*, *31*). Although these strategies perform well individually, few existing bioelectronic patches can simultaneously meet all requirements.

In this study, we introduce a glucose-powered electronic bandage (GEB) that integrates an electrospun polymer base, a self-assembled MXene (Ti_3_C_2_) conductive network, and an enzyme cascade to enable complete component degradation. An adhesive conductive hydrogel ensures mechanical compliance and stable electrical coupling with irregular wounds, whereas nanoconfinement strategies enhance catalytic stability and electron transfer for sustained bioenergy in diabetic environments. We systematically validated its therapeutic efficacy in diabetic mouse skin wounds, porcine full-thickness skin defects, and mouse intestinal injury model, where the device regulated cell fate, reprogrammed macrophage polarization, and promoted angiogenesis, achieving “electrical regulation–active repair.” Moreover, its material modularity and structural adaptability allow customized configurations for wounds of different types, sizes, and anatomical sites, demonstrating a promising platform for the intelligent evolution of next-generation bioelectronic dressings.

## RESULTS

### Design and mechanisms of the GEB

**Fig. 1.**
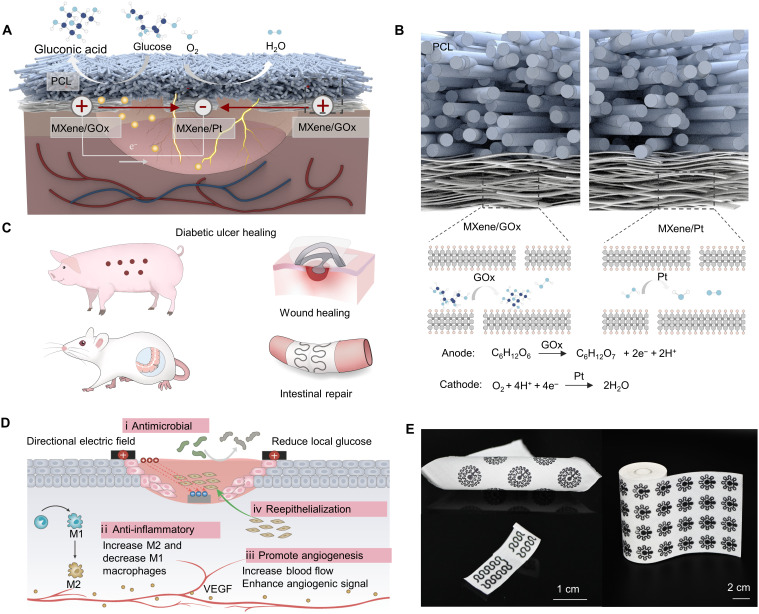
Schematics of the glucose-powered bioabsorbable soft electronic bandage for accelerated diabetic wound healing. (**A**) Schematic showing the glucose-powered electricity generation by the GEB to enhance the endogenous EF for wound healing. (**B**) Enlarged view of the cathode and anode with MXene nanoconfinement structure to boost enzymatic activity. (**C**) Application of the GEB on a diabetic ulcer wound, illustrating infected wound healing and intestinal tissue repair. (**D**) Mechanism of GEB-mediated healing through antibacterial, anti-inflammatory, reepithelialization, and angiogenesis. (**E**) Photograph of the GEB with customizable electrode shapes adapted to wound geometry.

The GEB is a thin, soft, and fully biodegradable dressing designed to provide self-sustained electrostimulation for diabetic wound healing. It consists of dual electrodes—MXene/glucose oxidase (MX-GOx) and MXene/Pt (MX-Pt)—that operate in concert to convert biochemical energy from wound exudate into a stable bioelectric field (Fig. 1A). The MX-GOx electrode functions as a bioresponsive anode, and when the MX-GOx electrode comes into contact with diabetic wound exudates, GOx catalyzes the oxidation of glucose to produce gluconic acid, protons, and electrons (*32*). The released electrons are transferred through MXene nanosheets to the MX-Pt electrode, where they drive the oxygen reduction reaction. Owing to the superior catalytic activity of MX-Pt, the oxygen reduction reaction (O_2_ + 4H^+^ + 4e^−^ → 2H_2_O) proceeds efficiently, enhancing the potential difference between the two electrodes (*33*). As a result, the system spontaneously generates a stable bioelectric field without the need for external energy input (Fig. 1B). This generated bioelectric field promotes fibroblast migration and proliferation, stimulates angiogenesis, accelerates tissue repair, and optimizes the wound healing microenvironment, leading to accelerated regeneration in diabetic skin ulcers and internal tissue injuries (Fig. 1, C and D).

The GEB is fabricated on an electrospun polymer substrate, onto which MX-GOx and MX-Pt electrodes are patterned using a mask-assisted spraying coating process (*34*) (Fig. 1E and fig. S1A). This scalable fabrication strategy allows for customizable electrode geometries and large-area production, whereas the electrospun polymer base ensures excellent flexibility, conformability, and biodegradability. Together, these features provide a practical pathway toward clinically adaptable, symbiotic bioelectronic dressings for comprehensive diabetic tissue regeneration.

### Structural and physical characterization

**Fig. 2.**
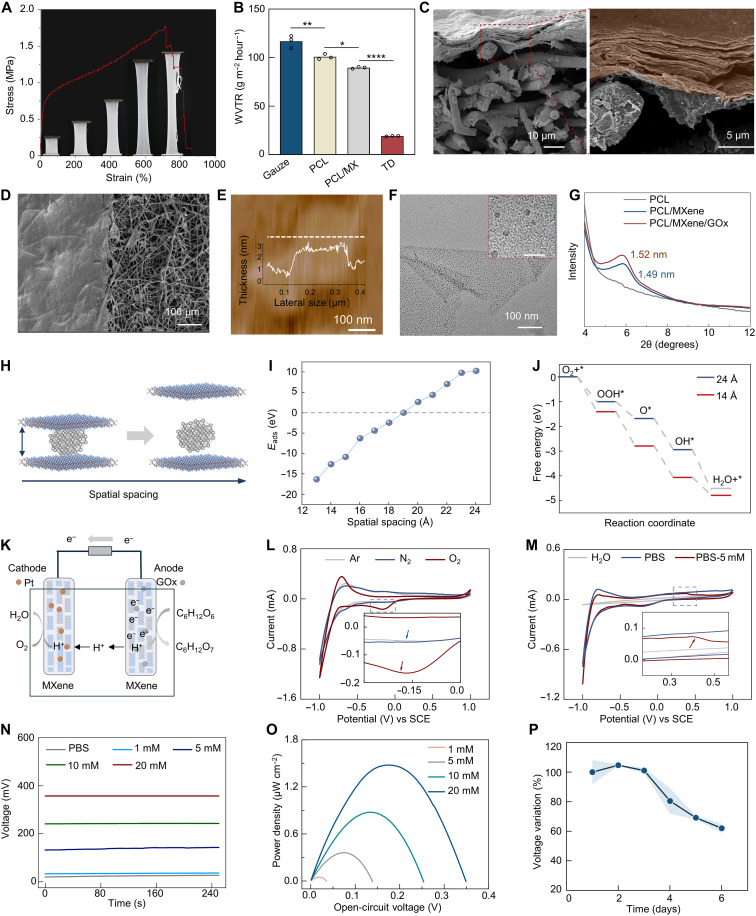
Basic performance characterization of the GEB. (**A**) Radial and axial stress-strain curves of PCL. (**B**) Comparison of the WVTR between the GEB and conventional wound dressings. (**C**) SEM image of the GEB interface showing the parallel-stacked structure of MXene. (**D**) Distinct boundary between sprayed MXene and the PCL substrate. (**E**) Piezoresponse force microscopy characterization and height profile of the MXene nanosheet. (**F**) Uniform distribution of Pt nanoparticles on the surface of MXene nanosheets. (**G**) XRD patterns of PCL and PCL/MXene composites. (**H**) Models of confined and nonconfined MXene/Pt structures. (**I**) Reaction pathway of oxygen reduction catalyzed by confined Pt nanoclusters. (**J**) Comparison of O_2_ adsorption energies on Pt sites under different confinement conditions. (**K**) Schematic illustration of enzymatic reactions at the cathode and anode. (**L**) CV curves of the cathode showing the catalytic activity toward O_2_ reduction. (**M**) CV curves of the anode showing the catalytic activity toward glucose oxidation. (**N**) Voltage outputs of the GEB in PBS solutions with different glucose concentrations. (**O**) Power density of the GEB. (**P**) Time-dependent voltage curves in solution environments.

Flexibility is crucial for wound dressings as excessive stiffness can damage regenerating tissues. Stress-strain analysis showed that the polycaprolactone (PCL) substrate exhibited a maximum strain of ~800% (Fig. 2A and fig. S1B), with an elastic modulus comparable to that of skin and commercial Tegaderm dressings, ensuring excellent conformability and effectively reducing stress concentration. The water vapor transmission rate (WVTR) of 89.5 g/m^2^·hour (Fig. 2B and fig. S1C) allows sufficient gas exchange while preventing excessive moisture accumulation and infection, thus providing a favorable microenvironment for cell migration and tissue repair (*35*).

Following MXene modification, the PCL surface exhibited a substantial increase in hydrophilicity, with the contact angle decreasing from ~99° to 19° (fig. S1D). This enhanced wettability facilitates absorption of wound exudate, promoting glucose diffusion to the catalytic interface and improving the efficiency of the glucose oxidation reaction. Scanning electron microscopy (SEM) revealed that pristine PCL formed a porous fiber network, whereas mask-assisted spraying coating produced a uniform MXene nanosheet layer on the fiber surface with clearly defined boundaries (Fig. 2, C and D), enabling precise patterning of electrodes with customizable geometries.

High-resolution characterization further highlighted the structural integrity and interface features of the MXene coating. Piezoresponse Force Microscopy (PFM) measurements indicated a nanosheet thickness of ~2 nm (Fig. 2E), ensuring efficient electron transport within the conductive network and shortening the electron transfer path from the enzymatic active site to the electrode, thereby reducing interfacial resistance. Energy-dispersive x-ray spectroscopy (EDX) confirmed uniform Ti distribution from the MXene (fig. S1E). Transmission electron microscopy (TEM) showed that platinum nanoparticles were evenly dispersed on the MXene surface without disrupting the layered structure (Fig. 2F), providing abundant catalytic sites to enhance oxygen reduction reaction kinetics. X-ray diffraction (XRD) revealed a characteristic peak at 6.3°, corresponding to an interlayer spacing of ~1.49 nm, which slightly increased to 1.52 nm after glucose oxidation (Fig. 2G), indicating that the MXene interlayer channels remain open during the reaction, facilitating dynamic exchange of glucose and reaction products and promoting enzyme-electrode synergistic catalysis.

Density functional theory (DFT) calculations were conducted to elucidate the nanoconfinement effect of MXene on the Pt-catalyzed O_2_ to H_2_O conversion. As shown in Fig. 2H, decreasing the interlayer spacing shortens the MXene/Pt distance, enhancing the confinement and increasing the contact area between O_2_ and active sites. The adsorption of O_2_ becomes progressively more stable with narrower channels, and when the spacing falls below 19 Å, the adsorption energy shifts from positive to negative, indicating that the confined environment provides energetic assistance for O_2_ activation (Fig. 2I). Reaction pathway analysis further shows that the 14-Å nanochannel exhibits a notably lower Gibbs free energy barrier for the complete four-electron reduction of O_2_ to H_2_O compared with the 2.4-nm system (Fig. 2J). Notably, the intrinsic interlayer spacing of MXene (1 to 2 nm) coincides with the optimal confinement range predicted by the simulations. Therefore, the nanoconfinement effect of MXene facilitates O_2_ activation and accelerates the overall reduction kinetics by optimizing the local catalytic microenvironment.

To evaluate the in vitro degradation behavior of the GEB, we performed a hydrogen peroxide (H_2_O_2_)–driven accelerated oxidative assay with inductively coupled plasma mass spectrometry (ICP-MS) analysis (fig. S2). In 0.1 M H_2_O_2_, the MXene-containing component underwent time-dependent oxidative degradation, with the electrode layer progressively oxidizing and dissolving into the solution. As degradation proceeded, the characteristic coloration gradually disappeared, and ultimately only the PCL fibrous substrate remained visible (fig. S2A). ICP-MS quantification further determined the elemental loading of the GEB (fig. S2B), with Ti at the milligram level (~2 mg, originating from MXene) and Pt at an ultralow microgram level (~0.9 μg). Li originated from LiF used during MXene processing and was only detectable near the instrumental detection limit (~0.8 ng per patch), indicating that Li-containing residues had been effectively removed during fabrication.

### Electrochemical performance

Building on its optimized architecture and favorable catalytic interfaces, the electrochemical characteristics of the GEB were systematically evaluated to assess its bioenergy conversion performance. The working principle of the GEB is illustrated in Fig. 2K. At the anode, GOx catalyzes the oxidation of glucose into gluconic acid and H_2_O_2_, followed by MXene-mediated H_2_O_2_ decomposition. The released electrons are transported through the conductive network to the cathode, where platinum catalyzes oxygen reduction to water, establishing a continuous redox cycle. Catalytic performance tests further confirmed the validity of these electrode reactions: The MX-GOx electrode markedly decreased the local pH during glucose oxidation (fig. S3A), whereas the MX-Pt electrode efficiently decomposed the generated H_2_O_2_ (fig. S3B), jointly maintaining charge balance.

Cyclic voltammetry (CV) analysis revealed characteristic electrochemical behaviors of the biofuel cell. Under an oxygen atmosphere, a distinct oxygen reduction peak appeared at −0.2 V (versus SCE), which weakened in air and disappeared under nitrogen (Fig. 2L), verifying the specific catalytic activity of the MX-Pt electrode toward oxygen reduction. On the anode side, a pronounced oxidation peak (~0.4 V) was observed in phosphate-buffered saline (PBS) containing 5 mM glucose, whereas no signal appeared in PBS (Fig. 2M), indicating efficient glucose oxidation catalyzed by MX-GOx. Both anodic and cathodic peak currents scaled linearly with the square root of the scan rate (fig. S3, C to F), demonstrating diffusion-controlled reaction kinetics (*36*).

With increasing glucose concentration, the electrochemical output of the GEB improved substantially. The open-circuit voltage increased from ~140 mV at 5 mM glucose to ~350 mV at 20 mM (Fig. 2N and fig. S4B), accompanied by a proportional rise in power density (Fig. 2O and fig. S4A) and total current density (fig. S4, C and D). Under physiological conditions (10 mM glucose, 37°C, pH 7.4), the biofuel cell operated stably for at least 6 days, maintaining over 60% of its initial voltage (Fig. 2P). These findings demonstrate that the GEB exhibits outstanding biocatalytic efficiency, steady electrochemical behavior, and long-term operational stability, thereby establishing a solid electrochemical foundation for glucose-powered self-sustained wound therapy.

### Biocompatibility and pro-angiogenic activity

**Fig. 3.**
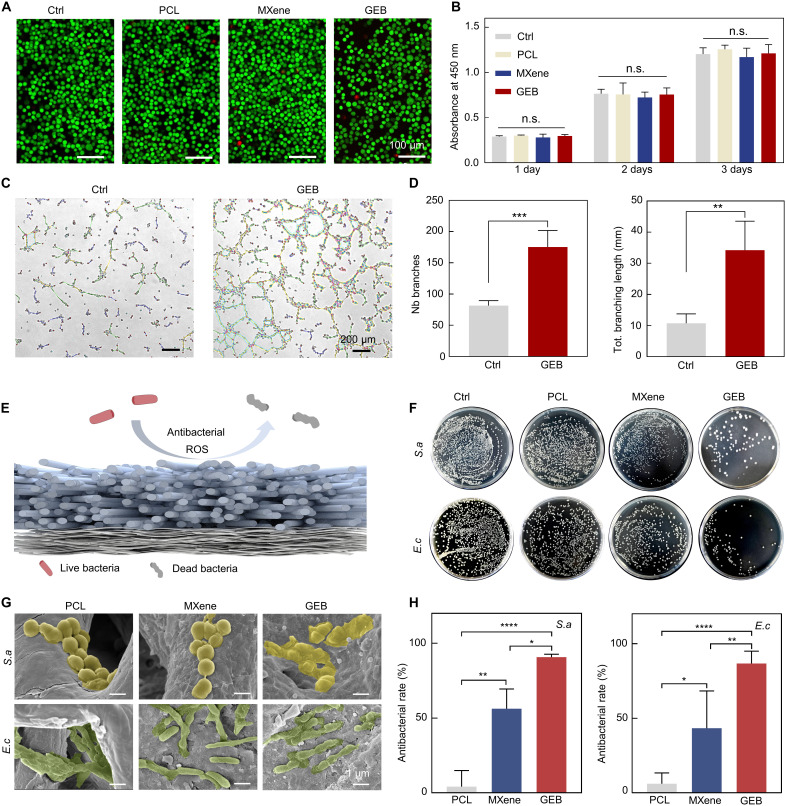
Cell biocompatibility and antibacterial performance of the GEB. (**A**) Live/dead cell staining of different materials. (**B**) CCK-8 assay to analyze cell viability. n.s., not significant. (**C**) Endothelial cell tube formation assay. (**D**) Corresponding Nb branching and total branching lengths in (C). (**E**) Schematic illustration of the antibacterial mechanism of the GEB. (**F**) Representative images of *E. coli* (*E.c*) and *S. aureus* (*S.a*) cell clumps cultured with different materials and the GEB. (**G**) SEM images of *S. aureus* (yellow) and *E. coli* (green) on PCL, MXene, and GEB. (**H**) Bacterial killing efficiency of different materials.

For diabetic wound healing, an ideal dressing must exhibit excellent biocompatibility to support cell adhesion, proliferation, and angiogenesis, thereby effectively promoting tissue regeneration. To evaluate this, the biocompatibility of the GEB was assessed. HaCaT cells were cultured on different materials, and live/dead staining (Fig. 3A) revealed that, after 3 days, cells in all experimental groups remained highly viable with no apparent cytotoxicity. Cytoskeletal staining further confirmed uniform adhesion and well-spread morphology of cells on the GEB surface (fig. S5, A and B), indicating that the material provides a favorable microenvironment for cellular growth. CCK-8 assays (Fig. 3B) showed that absorbance values of cells cultured for 1, 2, and 3 days were comparable to those of the control group and increased progressively over time, suggesting that the GEB does not inhibit cell proliferation and effectively supports cell growth. In addition, CCK-8 assays of MXene solutions at increasing concentrations demonstrated excellent cytocompatibility, with cell viability remaining comparable to the control even at 1 mg ml^−1^ (fig. S5C), further confirming the suitability of MXene for long-term biological applications in the GEB (*37*).

Building on these results, hemocompatibility was evaluated as clinical wound dressings must remain safe upon direct blood contact. Hemolysis assays (fig. S4D) indicated that the average hemolysis rates for the GEB, MXene, and PCL were all below 5%, far below the internationally accepted safety threshold, confirming excellent blood compatibility and biosafety (*38*).

The excellent cytocompatibility of the GEB not only ensures cellular safety but also provides a supportive microenvironment for angiogenesis. Therefore, we further assessed its pro-angiogenic potential using an in vitro tube formation assay with human umbilical cord endothelial cells (HUVECs) (Fig. 3C). Compared with the control group, endothelial cells cultured on the GEB formed a more continuous and intact tubular network within Matrigel, with significantly increased branch numbers (Nb branches), total branch length, and junction points (Nb junctions) (Fig. 3D and fig. S5E), and a trend toward increased average branch length (fig. S5F). These results indicate that the GEB markedly enhances endothelial angiogenic activity, ensuring adequate blood supply to support subsequent tissue repair and regeneration.

Given that the GEB incorporated trace inorganic components (MXene/Pt) intended for transient use, we next performed an initial in vivo fate assessment to complement the above in vitro biocompatibility evaluation. Elemental biodistribution was quantified by ICP-MS in Sprague-Dawley (SD) rats at 0 and 3 weeks after subcutaneous implantation to provide an initial in vivo assessment of the fate of MXene- and Pt-associated species (fig. S6A). Overall, Ti and Pt levels in blood and most major organs showed only a slight increase at 3 weeks without statistical significance. Notably, the kidney exhibited a significant elevation of Pt at 3 weeks (*P* < 0.01), consistent with a kidney-involved handling/clearance profile for trace Pt residues given its nanoscale form and ultralow total loading (fig. S6, B and C). In parallel, Li levels in blood and all examined organs remained essentially unchanged compared with preimplantation baselines (fig. S6D). This observation was consistent with the trace Li content in the patch measured in vitro, indicated that residual Li associated with MXene processing did not lead to appreciable in vivo distribution or accumulation.

### Antibacterial performance

Wound infection is a major factor that impedes the healing of diabetic wounds. Hyperglycemic microenvironments not only suppress host immune responses but also promote pathogen proliferation, leading to persistent infection, chronic inflammation, and delayed tissue repair (*39*). Therefore, dressings with potent antibacterial activity are critical for reducing infection risk and facilitating tissue regeneration (Fig. 3E). To systematically evaluate the antibacterial performance of the GEB, four coculture groups were designed: control (bacteria only), PCL + bacteria, MXene + bacteria, and GEB + bacteria. After 6 hours of coculture, bacterial suspensions were diluted and subjected to colony-forming unit (CFU) analysis (Fig. 3F), revealing that the GEB exhibited pronounced antibacterial activity against both *Escherichia coli* and *Staphylococcus aureus*.

SEM further revealed the morphological changes of bacteria attached to different materials (Fig. 3G). In the PCL and MXene groups, *S. aureus* cells maintained intact spherical structures with smooth surfaces and no apparent damage. In contrast, bacteria on the GEB exhibited pronounced surface shrinkage and membrane fusion, indicative of severe structural collapse. Similar morphological alterations were observed in *E. coli*, further confirming the superior antibacterial effect of the GEB. Quantitative analysis demonstrated that the antibacterial rates of PCL, MXene/PCL, and the GEB against *E. coli* were 6.7, 43.3, and 86.7%, respectively, and against *S. aureus* were 4.1, 56.3, and 90.7% (Fig. 3H), highlighting the remarkable broad-spectrum antibacterial activity of the GEB.

To elucidate the underlying mechanism, methyl violet solution was used as a reactive oxygen species (ROS) indicator and coincubated with the GEB. The solution gradually decolorized within 2 hours (fig. S6E), indicating ROS-mediated degradation. Time-dependent absorbance measurements further confirmed this process (fig. S5F). Collectively, these results demonstrate that the GEB catalyzes glucose oxidation to generate ROS, which disrupts bacterial membranes and induces cell death, thereby achieving potent antibacterial activity (*40*, *41*).

### Acceleration of wound healing by the GEB in diabetic mouse models

**Fig. 4.**
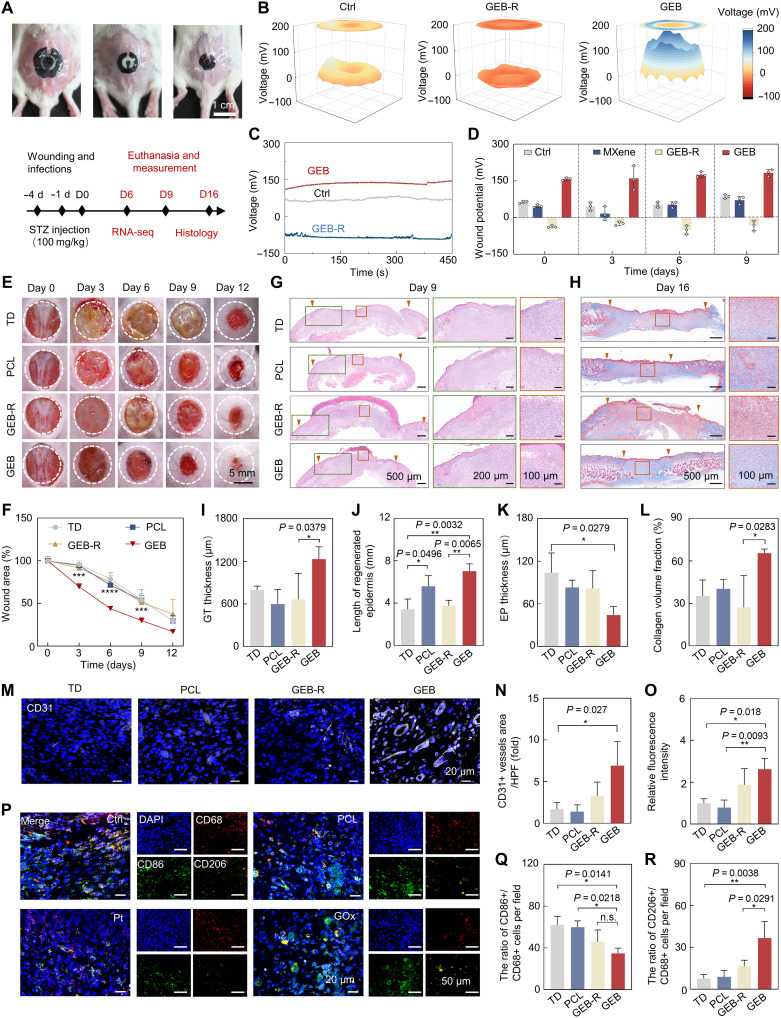
Repair of skin wounds in a diabetic mouse model. (**A**) Experimental images and schematic illustrations for skin wound repair in diabetic mice. d, days; RNA-seq, RNA sequencing. (**B**) Three-dimensional (3D) profile of the wound potential in different groups on day 0. (**C**) The potential of the wounds in different experimental groups remained stable over a certain period of time. (**D**) Monitoring of wound potential during the treatment process. (**E**) Photographs of the wound under different treatment modes. Scale bar, 5 mm. (**F**) Quantification of the open wound closure rate. (**G**) H&E staining of Kunming mice wounds on day 9. Three independent images were taken for each group. The red triangles marked wound margins. (**H**) Masson’s staining of porcine wounds on day 16. Three independent images were taken for each group. (**I**) Granulation tissue (GT) thickness in different groups. (*n* = 3). (**J**) Length of the regenerated epidermis in different groups (*n* = 3). (**K**) Epidermis (EP) thickness on day 16. (**L**) Collage volume fraction on day 16. (**M**) Immunofluorescence staining of CD 31 in different groups on day 9. (**N**) Quantitative analysis of CD31-positive staining on the ninth day. (**O**) Quantitative analysis of VEGFA-positive staining on the ninth day. (**P**) Immunofluorescence staining of CD68, CD86, and CD206 in different groups on day 9. (**Q**) Quantitative analysis of the ratio of CD86^+^/CD68^+^ cells in (P). (**R**) Quantitative analysis of the ratio of CD206^+^/CD68^+^ cells in (P).

To elucidate the therapeutic efficacy of the GEB in diabetic wound repair, an infected diabetic mouse model was established and treated with different dressings (*42*) (Fig. 4A). Mice with blood glucose levels exceeding 16.1mM were considered successfully modeled (fig. S7A). During the healing process, dressings of appropriate size were applied according to wound area, and the enzyme activity of GOx was found to scale proportionally with the dressing size (fig. S7B). The mice were randomly divided into four groups: transparent dressing (TD), PCL dressing, GEB-Reverse (GEB-R; GOx coated on the inner electrode), and GEB (GOx coated on the outer electrode). Wound surface potential mapping revealed that the GEB generated an “outer-positive/inner-negative” distribution pattern resembling the physiological wound field but with a higher amplitude, whereas GEB-R exhibited an overall negative potential (Fig. 4B). The average wound potentials in the GEB, PCL, and GEB-R groups were 140, 70, and −60 mV, respectively (Fig. 4C). These in vivo potential differences reflected a directional, endogenous dc EF oriented toward the wound center, with the corresponding output current at the microampere level. These bioelectric parameters fell within a reported effective window for promoting wound regeneration and tissue repair. Continuous monitoring further confirmed that the GEB maintained a substantially higher potential throughout the healing period (Fig. 4D), suggesting that it can sustain an enhanced bioelectric field to promote cell migration and tissue regeneration.

Wound closure analysis demonstrated that the GEB markedly accelerated healing (Fig. 4, E and F). On day 9, the residual wound area in the GEB group (29.9%) was significantly smaller than those in the TD (53.8%), PCL (53.8%), and GEB-R (51.1%) groups (Fig. 4F and fig. S7C). Hematoxylin and eosin (H&E) staining and Masson staining further revealed that GEB-treated wounds exhibited more complete reepithelialization, thicker granulation tissue, reduced inflammatory infiltration, and denser collagen deposition on days 9 and 16 (Fig. 4, G and H). Quantitative analyses showed significantly greater granulation thickness (*P* = 0.0379) and neoepithelial length (*P* = 0.0032) in the GEB group than in controls, with collagen volume fraction approaching that of normal skin (Fig. 4, I to L). These results indicate that the GEB effectively accelerates wound closure and tissue remodeling by reconstructing and amplifying the physiological EF at the wound site.

### GEB enhances angiogenesis and modulates the inflammatory microenvironment

Immunofluorescence staining provided further insight into the mechanism of enhanced tissue repair. As shown in CD31 and vascular endothelial growth factor A (VEGFA) staining (Fig. 4M and fig. S7D), the GEB group exhibited markedly higher microvessel density and VEGFA expression than other groups. Quantitative analyses confirmed significantly elevated CD31^+^ vessel density (*P* < 0.05) and up-regulated VEGFA levels in GEB-treated wounds relative to the TD and PCL groups (Fig. 4, N and O). Notably, the GEB-R group also showed increased vascularization compared with the TD and PCL groups, although the extent of neovascularization remained lower than that observed in the GEB group, indicating that glucose consumption–induced reduction in local blood glucose can, to some extent, promote angiogenesis, whereas the reversed EF partially impairs this pro-angiogenic effect. Together, these data suggest that the GEB establishes a highly pro-angiogenic microenvironment that ensures sufficient nutrient and oxygen supply for tissue regeneration.

Furthermore, immunofluorescence analysis of macrophage markers revealed that the GEB modulated macrophage polarization (Fig. 4P). Classically activated macrophages (M1) (CD68^+^/CD86^+^) are pro-inflammatory, whereas alternatively activated macrophages (M2) (CD206^+^/CD86^+^) facilitate inflammation resolution and tissue remodeling (*43*). The results showed a significant change in macrophage composition in the GEB-treated group. Specifically, the proportion of M1 macrophages decreased from 62% in the control group to 34.7% in the GEB-treated group, whereas the percentage of M2 macrophages increased from 7.6% in the control group to 36.6% in the GEB group (Fig. 4, Q and R, and fig. S7E). In contrast, no significant differences were observed between the PCL group and the control group. The GEB-R group exhibited fewer M1 macrophages and more M2 macrophages compared to the control group. However, this shift was less pronounced than in the GEB group, suggesting that glucose consumption and the resulting reduction in blood glucose levels might help alleviate the wound’s inflammatory state by increasing the proportion of M2 macrophages, potentially contributing to a more regenerative environment.

Peripheral blood analysis further corroborated these findings (fig. S8). In infected wounds, an elevated lymphocyte ratio typically signifies progression to the adaptive immune phase, beneficial for pathogen clearance and tissue repair. Both GEB and GEB-R groups showed increased lymphocyte and reduced neutrophil proportions on days 9 and 16, suggesting alleviated inflammation and improved immune homeostasis (*44*). Histological examinations of major organs at day 16 revealed no pathological abnormalities, inflammatory foci, or structural damage (H&E staining), confirming the excellent biocompatibility and systemic safety of the GEB (fig. S9A). Longitudinal monitoring of body weight revealed that, although all diabetic mice exhibited progressive weight loss, the decline was substantially attenuated in the GEB-treated group during the later stages of observation (fig. S9B). Concomitant measurements of blood glucose showed no significant changes on day 16 (fig. S9C), excluding glycemic reduction as a contributing factor. These findings suggest that the relative stabilization of body weight reflects an overall improvement in systemic physiological status, likely associated with accelerated wound repair and mitigated inflammatory burden.

The therapeutic mechanism of the GEB can be attributed to several synergistic effects. The MXene/GOx electrode catalyzes glucose oxidation and generates ROS, effectively suppressing bacterial growth and improving the local wound microenvironment. In addition, the MXene/GOx and MXene/Pt dual-electrode system provides localized ES, markedly enhancing cell proliferation, migration, and angiogenesis, thereby accelerating tissue repair and wound closure. Furthermore, the bioelectric field regulates the expression of inflammatory mediators, promotes macrophage polarization toward the M2 phenotype, attenuates excessive inflammatory responses, and optimizes the regenerative microenvironment, ultimately facilitating efficient tissue repair and remodeling.

### Transcriptomic analysis

**Fig. 5.**
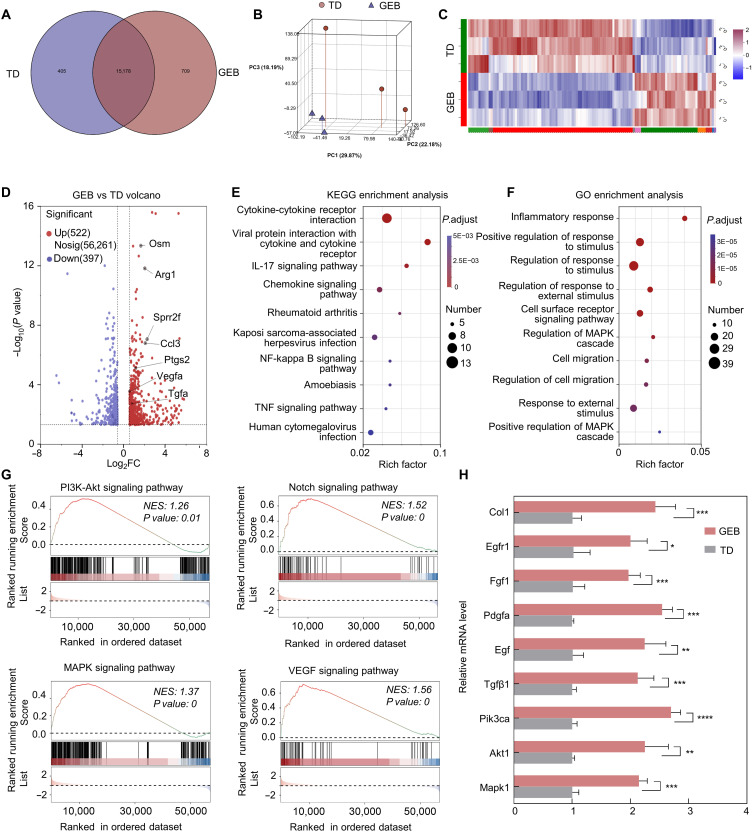
Transcriptome analysis of wound tissue on day 6. (**A**) Venn diagram showing the overlap of differentially expressed genes (DEGs) between the PCL and GEB groups. (**B**) PCA illustrating transcriptional differences among groups. (**C**) Heatmap of DEGs between the Ctrl and GEB groups. (**D**) Volcano maps of differential expression genes in the GEB group relative to the Ctrl group, with up-regulated genes (red dots) and down-regulated genes (blue dots). FC, fold change. (**E**) Bubble plots of Kyoto Encyclopedia of Genes and Genomes (KEGG) pathways enriched in DEGs from the GEB versus Ctrl comparison. (**F**) Bubble plots of GO pathways enriched in the DEGs from the GEB versus Ctrl comparison. (**G**) Gene set enrichment analysis (GSEA) of repair-related signaling pathways and biological processes. NES, normalized enrichment score. (**H**) Relative mRNA level in the diabetic wound tissue measured by real-time qPCR (*n* = 3 biologically independent samples per group).

To further elucidate the mechanism of GEB-mediated diabetic wound repair, transcriptomic sequencing was performed on wound tissues collected on postoperative day 6. Venn diagram and principal components analysis (PCA) (Fig. 5, A and B) revealed substantial transcriptional divergence between the GEB and TD groups, with 709 up-regulated and 405 down-regulated genes, indicating that the GEB markedly reprogrammed wound gene expression. The heatmap and volcano plot (Fig. 5, C and D) highlighted significant changes in key genes such as oncostatin M (*Osm*), arginase 1 (*Arg1*), small proline-rich protein 2f (*Sprr2f*), C-C motif ligand 3 (*Ccl3*), prostaglandin-endoperoxide synthase 2 (*Ptgs2*), vascular endothelial growth factor (*Vegf*), and transforming growth factor–α (*Tgfa*), which are involved in inflammation modulation, angiogenesis, epithelial proliferation, and extracellular matrix remodeling, suggesting a coordinated regenerative response induced by the GEB (*45*–*48*).

Pathway enrichment analyses (Fig. 5, E to G, and fig. S10, A and B) indicated that these genes were predominantly involved in the phosphoinositide 3-kinase–protein kinase B (*PI3K-Akt*), mitogen-activated protein kinase (*MAPK*), vascular endothelial growth factor (*VEGF*), Janus kinase–signal transducer and activator of transcription (*JAK-STAT*), Notch, and nuclear factor κB (*NF-κB*) pathways, as well as immune-related networks including cytokine-cytokine receptor interaction, tumor necrosis factor (*TNF*), nucleotide-binding oligomerization domain-like receptor (*NOD*-like receptor), *ErbB*, and rat sarcoma (*RAS*) signaling pathways. These pathways cooperatively regulate cell migration, neovascularization, epithelial regeneration, immune homeostasis, and extracellular matrix deposition, highlighting that the GEB orchestrates wound repair through coordinated activation of multiple regenerative signaling cascades rather than a single molecular route (*49*).

Furthermore, inflammatory gene heatmap analysis (fig. S10C) showed substantial down-regulation of pro-inflammatory mediators in the GEB group, indicating alleviation of excessive inflammation and restoration of immune balance. Gene Ontology (GO) annotation (fig. S10D) demonstrated enrichment in categories related to cellular processes, immune system regulation, metabolism, response to stimuli, and developmental processes, reflecting GEB’s broad role in cellular reprogramming and tissue homeostasis restoration.

Consistent with the transcriptomic findings, quantitative polymerase chain reaction (qPCR) validation confirmed significant up-regulation of mitogen-activated protein kinase 1 (*Mapk1*), protein kinase B (*Akt1*), phosphoinositide-3-kinase catalytic subunit alpha (*Pik3ca*), transforming growth factor–β1 (*Tgfβ1*), epidermal growth factor (*Egf*), platelet-derived growth factor alpha (*Pdgfa*), fibroblast growth factor 1 (*Fgf1*), epidermal growth factor receptor 1 (*Egfr1*), and collagen type I (*Col1*) (*P* < 0.05), key regulators of cell proliferation, angiogenesis, and collagen synthesis. Collectively, these findings demonstrate that the GEB catalyzes glucose oxidation to reduce local glucose concentration while providing sustained ES, synergistically activating the PI3K-Akt/MAPK/VEGF signaling axis to enhance cellular proliferation and angiogenesis, concurrently promoting M2 macrophage polarization, thereby reconstructing a proregenerative microenvironment that accelerates wound healing and skin barrier restoration.

### Acceleration of wound healing by the GEB in diabetic porcine models

To further assess the prohealing efficacy of the GEB under clinically relevant conditions and their translational potential, we performed wound healing experiments in diabetic porcine models. Porcine skin closely resembles human skin in anatomical structure, physiology, and healing mechanisms, making it a more faithful model for chronic refractory wounds (*50*). Throughout the study, blood glucose levels in the porcine remained elevated (fig. S11A), confirming successful induction of diabetes, whereas body weight monitoring (fig. S11B) showed a gradual decline within a physiologically acceptable range, consistent with typical diabetic characteristics.

**Fig. 6.**
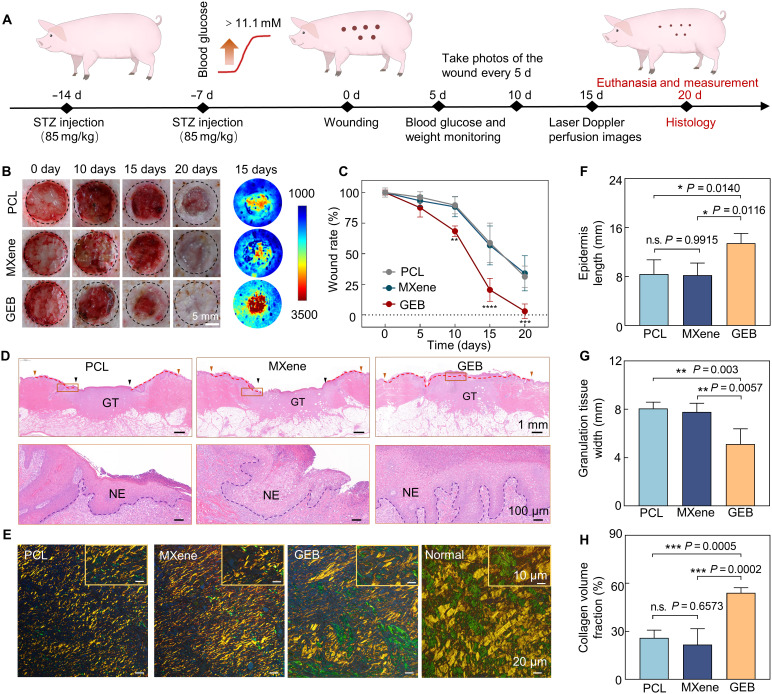
In vivo wound healing efficacy on diabetic Bama mini pigs. (**A**) Schematic of the in vivo study design. (**B**) Representative images of diabetic wounds of mini pigs in different treatment groups. (**C**) In vivo wound closure rates of pigs in different treatment groups. *n* = 3 biologically independent samples. (**D**) Representative H&E staining images of wounds in different treatment groups on day 20. The red triangles denote the wound margin, and the black triangles denote the newborn epidermis (NE). (**E**) Sirus red stain images of wounds in different treatment groups on day 20. (**F**) Quantitative analysis of the epidermis length of wounds in different treatment groups on day 20 (*n* = 3 biologically independent samples). (**G**) Quantitative analysis of the granulation tissue width of wounds in different treatment groups on day 15. (**H**) Quantitative analysis of the collage volume fraction in different treatment groups.

Standardized full-thickness skin defects (15 mm in diameter) were created on the dorsal region of diabetic pigs, and animals were assigned to three groups: PCL, MXene-loaded PCL (MXene), and GEB (Fig. 6A). Dressings were periodically replaced, and wound areas were photographed to evaluate healing progression. By day 10, wound area in the GEB group had decreased to 68%, compared with ~88% in the PCL and MXene groups, and by day 20, GEB-treated wounds were nearly completely closed, whereas PCL and MXene groups retained 31 and 34% of the initial wound area, respectively (Fig. 6, B and C). Blood flow assessment on day 15 revealed that the GEB group exhibited the highest perfusion rate, indicating enhanced angiogenesis and sufficient tissue perfusion to support accelerated wound repair. These findings collectively demonstrate that the GEB provides a sustained advantage in promoting wound closure throughout the healing process.

H&E staining (Fig. 6D) confirmed that, by day 20, wounds in the GEB group exhibited continuous and intact epidermis, whereas PCL and MXene groups still showed incomplete epithelial coverage and residual defects. Morphometric evaluation indicated that neoepidermal thickness in the GEB group approached that of normal skin, whereas epidermis in the PCL and MXene groups was thinner and infiltrated with inflammatory cells, reflecting slower repair. Sirius red and Masson’s trichrome staining (Fig. 6E and fig. S11C) further revealed denser and more organized collagen fibers in the GEB group, with markedly increased deposition compared with controls, closely resembling normal skin architecture. Quantitative analysis of epidermal length (Fig. 6F), granulation tissue width (Fig. 6G), and collagen volume fraction (Fig. 6H) confirmed more mature tissue regeneration in the GEB group, indicating superior repair efficacy relative to the control groups. Safety evaluations showed no pathological abnormalities in major organs across all groups (fig. S11D). In addition, routine blood parameters remained stable throughout the treatment period (fig. S11E), with no evidence of systemic infection or inflammatory responses, demonstrating excellent biocompatibility of the GEB.

These results demonstrate that the GEB exhibit remarkable therapeutic efficacy in diabetic porcine refractory wounds, not only accelerating wound closure and promoting angiogenesis and collagen deposition but also maintaining excellent biocompatibility throughout the treatment period, providing strong experimental support for their potential clinical translation.

### Intestinal wound repair in diabetic mouse models

**Fig. 7.**
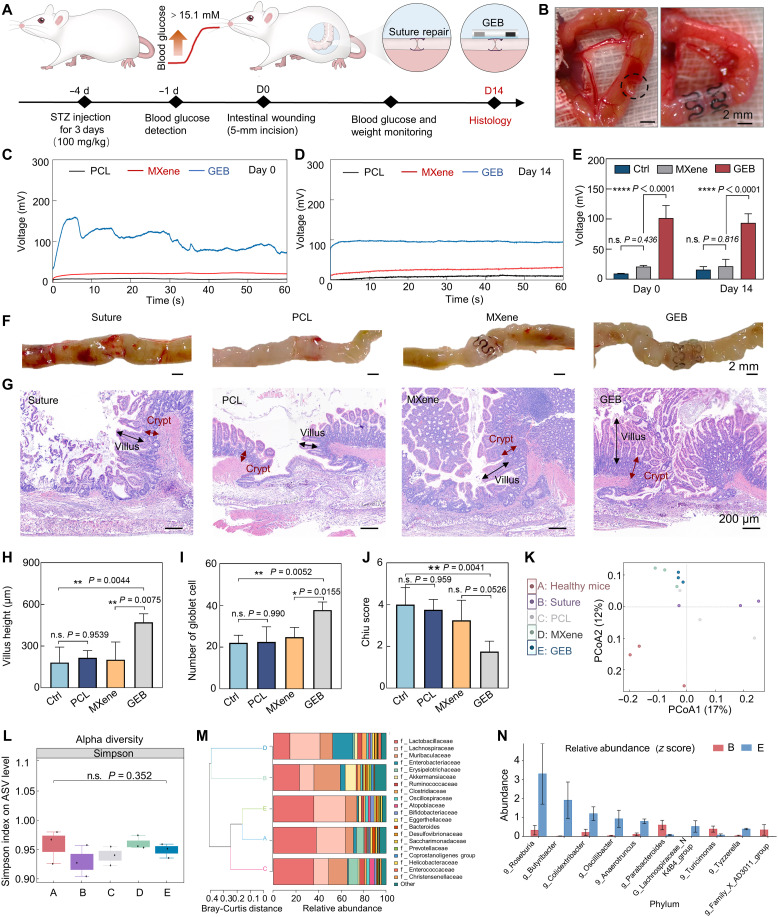
Repair of intestinal wounds in a diabetic mouse model. (**A**) Schematic illustration of in vivo intestinal defect-repair studies using sutures and the GEB. (**B**) Representative images of the intestinal defect-repair procedure, with images sourced from fig. S12A. (**C**) Measurement of intestinal electrical potential immediately after GEB implantation (day 0). (**D**) Measurement of the intestinal electrical potential 14 days after GEB implantation. (**E**) Quantification of the intestinal electrical potential. (**F**) Representative gross images of the intestine after 14 days of repair. (**G**) Representative H&E-stained histological images of intestinal defects repaired by sutures or the GEB after 14 days. (**H**) Quantification of goblet cells at the wound site. (**I**) Quantification of villus numbers at the wound site. (**J**) Histological Chiu’s score of intestinal injury. (**K**) PCA of the intestinal microbiota composition. (**L**) Microbial α-diversity based on amplicon sequence variant (ASV) was evaluated using the Simpson index. (**M**) Relative abundance of intestinal microbial families at the family level. (**N**) Relative abundance of intestinal microbial genera at the genus level.

Given the biodegradable nature of the GEB, we further evaluated its adaptability and therapeutic potential in vivo through diabetic intestinal wound repair, following the successful in vitro assessments in mouse and porcine skin wound models (Fig. 7A and fig. S12A). Four experimental groups were included: suture group, PCL group, MXene group, and GEB group. To accommodate the anatomical and mechanical characteristics of the intestinal wound and enable precise application in small-animal models, a parallel serpentine double-electrode bandage was designed (Fig. 7B). The bandage was coated with a gelatin methacryloyl (GelMA) hydrogel layer that enabled rapid and firm attachment to ~5-mm intestinal wounds within 10 s. In addition, we monitored the local glucose dynamics at the intestinal wound site during surgery and observed that, compared with the PCL group, the GEB group exhibited a rapid decrease in glucose concentration from ~22 to ~7 mM within 90 min (fig. S12B), whereas the PCL group showed little change, indicating that the device actively regulated the hyperglycemic wound milieu at the early stage of treatment.

The potential difference recorded between the two electrodes, as shown in Fig. 7C (day 0) and Fig. 7D (day 14), indicated that the PCL and MXene groups produced negligible voltage, whereas the GEB group generated an initial output of ~100 mV on day 0. The fluctuations observed at this early stage were mainly attributable to dynamic changes in the wound microenvironment. Immediately after surgery, variations in glucose concentration, together with alterations in local perfusion, exudate composition, and the adhesion and conductivity at the electrode-tissue interface, collectively contributed to the instability of the measured voltage. By day 14, the GEB remained firmly attached to the wound and improved mechanical integration with the surrounding tissue further stabilized the electrode-tissue interface. During this period, the glucose concentration in the wound area also became more stable, resulting in a markedly more consistent voltage output in the GEB group (Fig. 7D). The slight reduction in voltage relative to day 0 was mainly ascribed to the gradual degradation of the electrodes. Overall, these findings demonstrate that the GEB was able to sustain its electrical output throughout the wound healing process (Fig. 7E). All animals achieved complete wound closure without signs of leakage (Fig. 7F). In addition, no pathological abnormalities were observed in the major organs (fig. S12C), confirming the safety of the GEB treatment. To further assess the therapeutic outcomes, histological analyses and gut microbiota profiling were conducted after 14 days of treatment and compared among all experimental groups.

Villus height (VH), a key indicator of mucosal injury and regeneration, was used to evaluate epithelial recovery. Histological analysis revealed that the suture group exhibited sparse and irregular villi with incomplete epithelial coverage, indicating poor mucosal integrity (Fig. 7G). Quantitative analysis confirmed a significant reduction in VH in this group (Fig. 7H). The PCL and MXene groups showed only marginal improvements as the villi remained disorganized and VH values were not significantly elevated, implying that these materials alone were insufficient to support efficient mucosal regeneration. In contrast, the GEB group exhibited well-organized villi with restored morphology, increased villus density, and continuous epithelial coverage. Notably, the number of goblet cells—critical for mucus secretion and mucosal protection—was markedly elevated in the GEB group compared to the other groups, with a more homogeneous distribution along the villi (Fig. 7I), suggesting accelerated goblet cell migration and restoration of secretory function. Further evaluation using the Chiu scoring system demonstrated that the suture group presented extensive epithelial detachment and the highest injury scores, whereas the PCL and MXene groups exhibited comparable levels of mucosal damage (*51*). By contrast, the GEB group displayed significantly lower injury scores (Fig. 7J). These results demonstrate that GEB treatment effectively enhances villus epithelial regeneration, accelerates goblet cell recovery, and promotes functional restoration of the intestinal mucosal barrier, thereby facilitating the structural and functional healing of intestinal wounds in diabetic mice.

To investigate the impact of GEB treatment on the intestinal microecological environment, 16*S* ribosomal DNA (rDNA) sequencing was performed. Comparison of microbial compositions among healthy mice (A), suture (B), PCL(C), MXene (D), and GEB (E) groups revealed distinct microbial community structures across groups, as evidenced by principal coordinate analysis (PCoA) and Venn diagrams showing tight intragroup clustering and clear intergroup separation (Fig. 7K and fig. S13A). α-Diversity analysis indicated no significant differences in species richness or evenness among the groups (Fig. 7L), suggesting that none of the treatments disrupted the overall intestinal microbial stability. Analysis of relative abundances at the class and family levels demonstrated that the microbiota composition of normal mice was most similar to that of the GEB group (Fig. 7M and fig. S13B), indicating that the GEB treatment helped restore the impaired intestinal microecological structure in diabetic mice with wounds.

Further analysis at the genus level revealed that, compared with the suture group, the GEB group exhibited a more balanced ratio of beneficial to potentially harmful bacteria (Fig. 7N). Specifically, butyrate-producing genera such as *Roseburia*, *Butyricicoccus*, *Collinsella*, and *Oscillibacter* were significantly enriched in the GEB group. These taxa are closely associated with short-chain fatty acid (SCFA) metabolism, mucosal energy supply, and epithelial repair (*52*). The moderate increase in beneficial bacteria in the GEB group suggests that this treatment promotes the proliferation of microbial communities related to energy metabolism and mucosal regeneration while maintaining overall microbial diversity, thereby preserving gut microbial stability and driving the intestinal microecology of diabetic mice toward a healthier and more balanced state.

## DISCUSSION

In summary, we developed a biodegradable, GEB that leveraged glucose metabolism to restore impaired endogenous EFs and promote diabetic wound repair. The bandage integrated antibacterial activity, glucose-lowering effect, and sustained ES delivery. These coordinated effects jointly guided cell migration, reprogram macrophage polarization, and promoted angiogenesis, thereby helping overcome key barriers to diabetic wound healing.The safety and efficacy of this strategy were validated across species (mice and pigs) and across organs (skin and intestine) in multiple preclinical models, including diabetic murine wounds, porcine full-thickness skin defects, and a mouse intestinal injury model, supporting its generalizability and translational potential. This energy conversion–driven platform also suggested broader applicability of degradable bioelectronics to other regenerative settings in which disrupted bioelectric signaling is a pathological hallmark, such as peripheral nerve injury and bone defect.

Relative to existing glucose fuel cell–based wound devices, the advantages of the GEB were not defined by maximizing any single electrochemical metric but by a clinically aligned integration that prioritized dressing-oriented attributes and translatability (table S2). First, the system was conceived as an implantable, fully biodegradable, short-time therapeutic dressing, thereby differing from nonresorbable system, and better matching clinical scenarios, particularly for internal deep or irregular wound, in which device removal after the therapeutic window is undesirable. Second, the GEB was realized as an ultrathin, breathable, and highly compliant soft electronic textile, and this architecture facilitated reliable coupling on irregular wounds and even curved, dynamic tissue surfaces. Third, the fabrication route was built on a mask-assisted spraying process, which naturally supported area scaling, batch replication, and rapid customization of shape and size and thus aligned more closely with large-scale manufacturing requirements. Notably, this short-term bandage positioning was further supported by the material selection: Both the polymer substrate and MXene were chosen for their biodegradability. In contrast, although Pt nanoparticles did not undergo “chemical degradation” in the conventional sense, their presence in the device at ultralow loading and in nanoparticulate form (~10 nm) increased the likelihood of recognition and uptake by macrophages and other phagocytes, potentially enabling gradual clearance through reticuloendothelial system-related routes and thereby reducing the risk of long-term retention. During the wound-healing observation period defined in this study, we did not detect overt local or systemic toxicity signals attributable to these inorganic components, collectively supporting the feasibility of the GEB as a short-duration, biocompatible electronic dressing.

It should be acknowledged that the long-term in vivo transformation and clearance of the inorganic components had not yet been quantitatively characterized over extended follow-up periods and that the effective operational lifetime of the enzymatic glucose fuel cell had not been systematically established under clinically relevant, complex conditions. Accordingly, future studies should develop more rigorous frameworks for long-term fate and safety assessment, together with standardized evaluations of output decay and device lifetime, to more clearly delineate performance boundaries and inform material iteration for indications requiring longer treatment windows.

## MATERIALS AND METHODS

### Fabrication of the GEB

PCL was dissolved in dichloromethane (15% w/v) and electrospun into fibrous mats using a 5-ml syringe with a 23-gauge needle at a flow rate of 1.5 ml hour^−1^ and a collector distance of 10 cm (*53*). During electrospinning, the collector reciprocated over a 10-cm range at 20 cm min^−1^, with applied voltages of +7 and −3 kV and a rotation speed of 300 rpm for 120 min. The obtained fibers were vacuum dried overnight at room temperature and subsequently plasma treated to remove surface contaminants and introduce hydrophilic groups, thereby improving electrode adhesion. MXene (Ti_3_C_2_T*_x_*) nanosheets were synthesized by selectively etching the Al layer from Ti_3_AlC_2_using a LiF/HCl system (2 g of LiF and 30 ml of HCl, diluted to 40 ml) at 45°C for 24 hours under stirring. The product was washed by repeated centrifugation (3500 rpm, 3 min per cycle) until the supernatant reached pH ≈ 6, redispersed in deionized water (60 ml), and ultrasonicated in an ice bath for 1 hour to achieve full delamination. A 1-mm-thick acrylic mask was laser cut to define electrode patterns. MXene/Pt and MXene/GOx hybrid inks were prepared by adding 100 μl of a platinum nanoparticle solution (1 mg ml^−1^) or 10 mg of GOx to 1 ml of MXene dispersion (20 mg ml^−1^), respectively. Using the patterned mask, the hybrid inks were spray coated onto plasma-treated PCL fibers, with MXene/Pt and MXene/GOx assigned to the outer and inner electrode regions, respectively, yielding two configurations: GEB (GOx outer, Pt inner) and GEB-R (GOx inner, Pt outer). Last, a thin MXene overlayer (20 mg ml^−1^) was spray deposited to interconnect the electrodes and encapsulate the enzyme layer, completing the fabrication of the GEB.

### Structural and mechanical characterization

The morphology and elemental distribution of the electrospun nanofibers were examined using a Nova 450 field-emission scanning electron microscope coupled with a Raith/EDAX EDX detector. The mechanical behavior of the fibrous mats was assessed by tensile testing on an ESM301/Mark-10 system, from which the corresponding stress-strain profiles were obtained.

### Electrical and electrochemical performance characterization

Electrical output characteristics, including voltage and current signals of the electronic textiles, were recorded using a Keithley 6517B electrometer in conjunction with a Teledyne LeCroy HD 4096 oscilloscope. The current-voltage (*I*-*V*) curves were further analyzed using a Keithley 4200 semiconductor parameter analyzer. All electrochemical measurements were performed using a CHI760E electrochemical workstation. CV measurements were carried out to evaluate the catalytic performance of the anode and cathode toward glucose oxidation and oxygen reduction, respectively. In the three-electrode configuration, the tested electrode served as the working electrode, a platinum foil acted as the counter electrode, and a saturated calomel electrode (SCE) was used as the reference. For anode characterization, CV scans (10 mV s^−1^, three cycles) were performed in PBS both in the absence and presence of 5 mM glucose. For cathode testing, CV scans (10 mV s^−1^, three cycles) were conducted in PBS under continuous purging with N_2_, O_2_, or air for 15 min before measurement, with gas flow maintained during scanning. Linear sweep voltammetry (LSV) was used to assess full-cell performance, where the cathode and anode were connected as working and combined counter/reference electrodes, respectively. LSV curves were obtained at a scan rate of 5 mV s^−1^ in PBS containing various glucose concentrations (1, 5, 10, and 20 mM) to analyze the correlations among glucose concentration, current density, open-circuit voltage, and power density.

### Full-thickness diabetic mouse wound model

Male Kunming mice (6 to 8 weeks) were fasted for 14 hours and intraperitoneally injected with 1 wt % streptozotocin (STZ; 100 mg/kg per day) for 3 consecutive days. Blood glucose was measured on day 4, and levels ≥16.1 mM indicated successful diabetes induction. Mice were subsequently provided 10 wt % sucrose waters. One week later, full-thickness dorsal skin wounds (10 mm in diameter) were created with a skin punch and randomly assigned to four groups (*n* = 6 per group): Ctrl (Tegaderm dressing), PCL (PCL bandage), GEB-R (GOx electrode at the inner position), and GEB (GOx electrode at the outer ring position). All wounds were covered with Tegaderm to prevent skin contraction. All procedures were approved by the Animal Ethics Committee of the Beijing Institute of Nanoenergy and Nanosystems (2022018LZ). Wound healing was monitored and measured every 3 days until complete closure. Wound images were captured at each time point, and healing rates were calculated using the ImageJ software.

### Full-thickness diabetic porcine wound model

Bama mini pigs (18 to 22 kg) were used under sterile conditions with veterinary supervision and ethical approval (NNXMJS001-20241112P4-1P-SY). After a 16-hour fast, STZ [prepared in 0.1 M sodium citrate buffer (pH 4.2 to 4.5), 0.22 μm filtered] was intravenously injected at 85 mg/kg via an ear vein catheter under anesthesia. Diabetes was confirmed by fasting blood glucose (>11.1 mM) for 2 consecutive days, with high-glucose feed provided 6 hours post-STZ to prevent hypoglycemia. Blood glucose was monitored daily, and subcutaneous insulin (12 to 20 U) was administered if levels exceeded 20 to 25 mM.

Under anesthesia (Zoletil 100 and xylazine hydrochloride), dorsal hair was removed, and six full-thickness circular wounds (1.5 cm) were created bilaterally along the spine. Wounds were randomly assigned to three groups: PCL, MXene (MXene-coated PCL), and GEB (GOx electrode at the outer ring position). Bandages were replaced every 3 days, with body weight, blood glucose, and wound images recorded regularly. On day 15, perfusion of regenerated skin was assessed via laser Doppler imaging (MoorFLPI-2, Moor Instruments, UK). On day 20, pigs were euthanized, and wound tissues were harvested for H&E, Masson’s trichrome, and Sirius red staining to evaluate microstructure and collagen deposition.

### Repair of intestinal wounds in a diabetic mouse model

A total of 24 male Kunming mice were intraperitoneally injected with STZ to induce diabetes and randomly divided into four groups (*n* = 6 per group): suture, PCL, MXene, and GEB groups. Under isoflurane anesthesia, abdominal hair was removed, and mice were placed on a 37°C heating pad. Following a midline laparotomy, intestines were exposed and separated using moist sterile gauze. A 5-mm longitudinal incision was made perpendicular to the intestinal axis, the incision was first closed with 7-0 absorbable surgical sutures, and then bandages coated with SunP Gel G1 (55 to 65% substitution) were applied to enhance adhesion to the intestinal surface and irradiated with ultraviolet (UV) for 15 s. Sealing was verified by injecting PBS into the lumen with a 32-gauge needle. Intestines were repositioned, and the abdominal wall was closed with 4-0 sutures. Body weight and complete blood counts were monitored on postoperative days 1, 3, 7, 11, and 14. On day 14, mice were euthanized, and a 5-mm intestinal segment centered on the wound was collected for histopathological analysis. For histology, H&E-stained sections (*n* = 6 per group) were examined under an inverted fluorescence microscope, and intestinal mucosal injury was graded using the Chiu scoring system (0 to 5) based on villus morphology, and goblet cells were quantified in five crypt-villus units per field. Intestinal contents (~5 g) were snap frozen at −80°C for gut microbiota sequencing. All procedures were approved by the Animal Ethics Committee of the University of Chinese Academy of Sciences (2025001HO).

### Statistical analysis

Statistical analyses were performed with the Origin software program, Prism 8.0 software (GraphPad Software), and ImageJ software, and the results were reported as the means ± SD. Analysis of significance difference was calculated by using the one-way analysis of variance (ANOVA) or unpaired Student’s *t* test. Statistical differences were shown with four significance levels (*P < 0.05, **P < 0.01, ***P < 0.001, and ****P < 0.0001).

## Supplementary Materials

### This PDF file includes:

Supplementary Materials and Methods Figs. S1 to S13 Tables S1 and S2 References
