# Supplementary material for: Restoration of endogenous electric fields with a glucose-powered symbiotic bioabsorbable bandage for diabetic wound healing
Source: Sci Adv. 2026 Jun 10;12(24):eaed9445. doi: 10.1126/sciadv.aed9445 (PMC13251872; doi:10.1126/sciadv.aed9445)
Supplement: Supplementary file 1 — Supplementary Materials and Methods Figs. S1 to S13 Tables S1 and S2 References [file sciadv.aed9445_sm.pdf]

Science Advances
AAAS

Supplementary Materials for

Restoration of endogenous electric fields with a glucose-powered symbiotic bioabsorbable bandage for diabetic wound healing

Lingling Xu *et al.*

Corresponding author: Engui Wang, wangengui@ucas.ac.cn; Chunying Chen, chenchy@nanoctr.cn; Zhou Li, li_zhou@tsinghua.edu.cn; Han Ouyang, ouyanghan@ucas.ac.cn

Sci. Adv. **12**, eaed9445 (2026)
DOI: 10.1126/sciadv.aed9445

This PDF file includes:

Supplementary Materials and Methods
Figs. S1 to S13
Tables S1 and S2
References

Supplementary Materials and Methods

Computational Methods

The density functional theory (DFT) within the projected augmented wave (PAW) method has been employed, as implemented in the Vienna ab initio simulation package (VASP) *(54)*. The generalized gradient approximation (GGA) of Perdew, Burke and Ernzerhof (PBE) was used for the electron exchange-correlation functional *(55)*. Full relaxation of the structures was performed by the conjugate gradient scheme until the maximum force on each atom was less than 1 meV $Å^{-1}$, and the total energy was converged to 10-5 eV. A kinetic energy cutoff of 450 eV is used for the plane-wave basis, and a 11 × 11 × 1 k-point mesh is adopted for the Brillouin zone sampling in the calculations.

Methyl violet degradation

PCL and GEB samples were incubated in 1 mL of methyl violet solution (50 μM) at room temperature. At 0.5 h intervals, aliquots were collected for photographic and absorbance measurements to monitor degradation kinetics.

Cell culture

Human umbilical vein endothelial cells (HUVECs) and immortalized human keratinocytes (HaCaTs) were obtained from the Chinese Academy of Sciences Cell Bank (Beijing). HaCaTs were cultured in RPMI-1640 medium with 10% FBS and 1% penicillin/streptomycin, and HUVECs in high-glucose DMEM with 10% FBS and 1% penicillin/streptomycin. Cells were maintained at 37°C in a 5% CO_2 incubator, with medium changes every two days and passaging at ~80% confluency.

Cell proliferation and viability (CCK-8)

PCL and GEB were cut into 3 cm^2 pieces, sterilized under UV light, immersed in 75% ethanol for 3 min, and rinsed three times with PBS. The samples were then soaked in 1 mL of complete culture medium at 37 °C for 24 h to obtain extract solutions. HaCaT cells were seeded in 96-well plates and cultured with the extracts, while cells in fresh medium served as controls. Cell viability was determined on days 1, 2, and 3 using the CCK-8 assay. At each time point, the medium was replaced with fresh medium containing 10% (v/v) CCK-8 reagent and incubated for 1 h. The absorbance of 100 μL aliquots at 450 nm was recorded using a microplate reader to quantify viable cells *(56)*.

Live/Dead cell staining

Material extracts were prepared as described above. HaCaT cells were incubated with the extracts for 24 h, then harvested, centrifuged, and resuspended in Calcein-AM solution. After incubation at 37 °C for 25 min in the dark, propidium iodide (PI) solution was added and cells were further incubated for 5 min at room temperature. The cells were washed with PBS and immediately imaged under a confocal laser scanning microscope. Live and dead cells emitted green and red fluorescence, respectively.

Cell morphology and cytoskeleton detection

PCL and GEB patches were cut into 1 cm diameter discs, sterilized under UV light, immersed in 75% ethanol for 3 minutes, and rinsed three times with PBS. They were then placed in 24-well plates and secured with PDMS rings (7 mm inner diameter) to

prevent floating, glass coverslips served as controls. Cells were fixed with 4% paraformaldehyde for 10 minutes and washed three times with PBS. They were then incubated with rhodamine-phalloidin (Abcam, diluted 1:1000) at 37 °C for 1 hour. After three PBS washes, cells were stained with Hoechst solution (Thermo, diluted 1:1000) at 37 °C for 10 minutes. Samples were washed in PBS, mounted under glass coverslips, and imaged via confocal microscopy.

Antibacterial assay

The antibacterial activity of sterilized PCL and GEB samples (1 cm in diameter) was tested against Staphylococcus aureus (29213) and Escherichia coli (ATCC 25922). Bacterial suspensions (2–3 × 10^4 CFU/mL) were incubated with the samples in 1 mL medium for 6 h. Afterward, the suspensions were serially diluted, plated on agar, and incubated for colony counting. For morphological analysis, bacteria-adhered samples were fixed in 2.5% glutaraldehyde, dehydrated with graded ethanol, dried, and observed by SEM.

Cell migration and angiogenesis assay

Matrigel matrix was thawed at 4℃ and homogenized with pre-cooled pipette tips. Pre-chilled 24-well plates were loaded with 20 μL Matrigel per well, spread evenly, and air-bubble-free. All procedures were performed on ice. Plates were sealed and stored at 4 ℃ overnight to allow gel spreading. Before cell seeding, plates were incubated at 37 ℃ for 45 minutes to facilitate matrix crosslinking. HUVECs were detached, resuspended in DMEM with 10% FBS, and seeded at 500 μL/well (four replicates). Patches were placed in PBS solution and incubated at 37 ℃, with images captured every 2 hours. Images were analyzed using ImageJ to quantify the number of branch points (Nb), total branch length (Tot. Branch Length), and average branch length.

Hemolysis test

Material films were immersed in PBS (6 cm^2/mL) and incubated at 37 ℃ for 24 hours to prepare extracts. Deionized water (positive control) and PBS (negative control) were used alongside PCL and GEB experimental groups. Fresh blood from male rats was anticoagulated and diluted 1:10 in PBS. Then, 100 μL of diluted blood was added to 1 mL of each sample (n = 4 per group) and incubated at 37 ℃ for 60 minutes. Samples were centrifuged at 1000 × g for 5 minutes, and supernatant absorbance was measured at 545 nm (reference: 655 nm) to calculate hemolysis rates *(57)*.

ICP-MS study

Inductively coupled plasma mass spectrometry (ICP-MS, Thermo iCAP Q) was used to quantify Ti, Li, and Pt in order to determine the elemental content of the GEB and to assess the fate of its trace inorganic residues *(58)*. In an in vitro accelerated oxidative degradation assay, GEB patches were immersed in 5 mL of 0.1 M H_2O_2 to model oxidative-stress conditions and accelerate degradation of the MXene-containing component. After 90 min, when MXene had fully degraded, the post-incubation solutions were collected for elemental analysis. For in vivo biodistribution, GEB patches were sterilized by ultraviolet irradiation and 75% ethanol treatment for 1 h and implanted subcutaneously in the dorsal region of Sprague–Dawley (SD) rats. At designated time points post-implantation, animals were euthanized and blood and major organs were harvested. Excised tissues were transferred to glass vessels and digested in concentrated HNO_3 overnight, followed by heating at 180 °C for 1 h. H_2O_2 was then added stepwise until complete digestion, and the digests were heated to 260 °C to

remove residual HNO_3. The resulting colorless solutions were diluted to 3 mL with 2% (v/v) HNO_3 and analyzed on an ICP-MS system.

In vivo electrical testing

Wound potential and current were measured using an electrometer, oscilloscope, and platinum electrodes. The positive and negative terminals of the electrometer were connected to two platinum electrodes spaced 1 mm apart. The potential difference on the wound surface was recorded to characterize the average electric field intensity of the region.

Histology and immunofluorescence

Wound tissues were photographed, excised, and fixed in 4% paraformaldehyde, followed by dehydration and paraffin embedding. Tissue sections (4 μm) were stained with hematoxylin and eosin (H&E), Masson's trichrome, and Sirius Red for histological evaluation. For immunofluorescence staining, sections were incubated with primary antibodies against CD31 (1:200, Abcam, ab28364), VEGFA (1:500, Proteintech, 66828-1-Ig), CD68 (1:200, Abcam, ab125212), CD86 (1:300, Santa Cruz, sc-52448), and CD206 (1:300, Santa Cruz, sc-58986), followed by appropriate fluorescent secondary antibodies. Nuclear counterstain was 4′,6-diamidino-2-phenylindole (DAPI) (Abcam, ab285390). Quantitative analysis of histological and immunofluorescence images, including wound closure, granulation tissue thickness, neoepidermal formation, and collagen deposition, was performed using ImageJ software.

Transcriptome sequencing and data analysis

Wound tissues were collected on day 6 post-injury for RNA extraction using TRIzol reagent. RNA quality was assessed with a 5300 Bioanalyzer, and concentration was measured using a NanoDrop ND-2000. RNA purification, reverse transcription, library preparation, and sequencing were performed by Majorbio (Shanghai, China) following the manufacture protocols. Sequencing was conducted on the NovaSeq X Plus platform (PE150, Illumina). Differential gene expression analysis was performed using DESeq2, with $|log2FC| \geq 1$ and $FDR < 0.05$ considered significant. GO enrichment and KEGG pathway analyses were conducted using Goatools and Python scipy packages.

qPCR analysis

Total RNA extracted from wound tissues was quantified and assessed for purity using a NanoDrop spectrophotometer at 260/280 nm wavelengths. RNA was then reverse-transcribed into cDNA using the QuantiTect Reverse Transcription Kit (Vazyme), qPCR reactions were prepared with cDNA templates, specific primers, Tag Pro Universal SYBR qPCR Master Mix (Vazyme), and reaction buffer, and performed using a thermal cycler. Relative gene expression was calculated using the Ct method with β-actin as the reference gene. Primer sequences are listed in Table S1.

Supplementary Figures

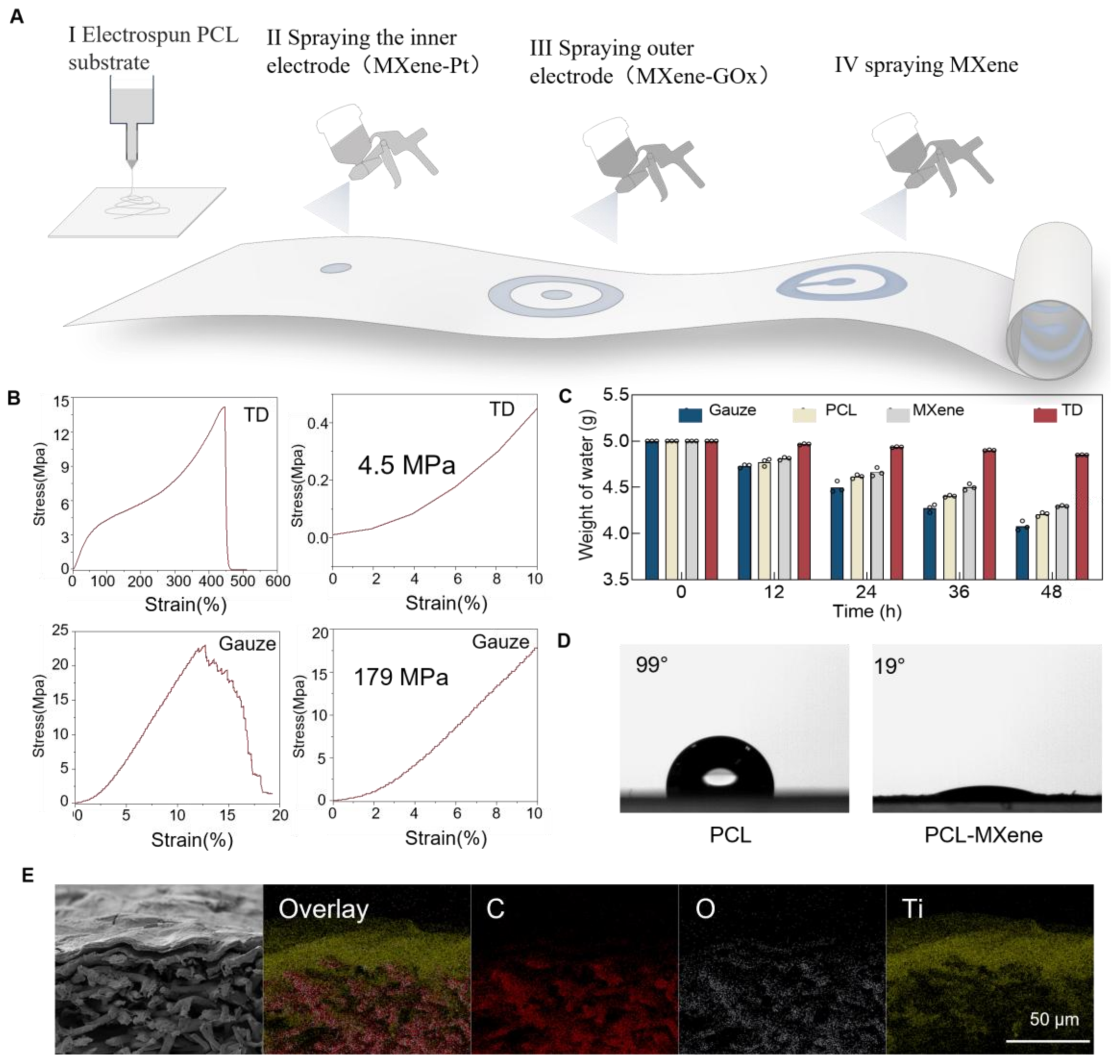

Fig. S1. Supplementary structural and physical characterization of the GEB components. (**A**) Illustration of the mask-assisted spraying coating process for fabricating MXene-modified electrodes on PCL. (**B**) Stress–strain curve of the PCL substrate showing a maximum strain of ~800%. (**C**) Water vapor transmission rate (WVTR) of different dressing. (**D**) Contact angle measurement showing enhanced hydrophilicity of PCL after MXene modification. (**E**) EDS mapping showing Ti distribution on MXene-coated PCL.

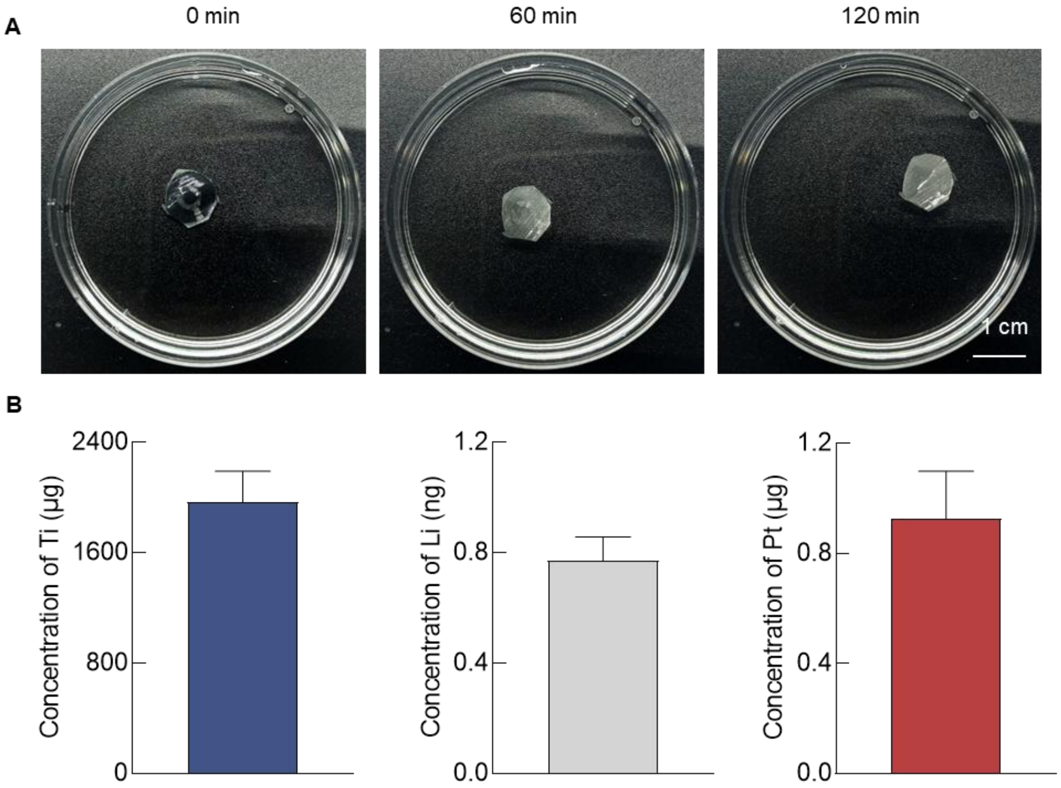

Fig. S2 In vitro accelerated degradation of the GEB. (**A**) Representative photographs showed that the GEB had undergone accelerated degradation in 0.1 M H_2O_2 solution over time (scale bar, 1 cm). (**B**) ICP-MS quantification had determined the total loading of key elements (Ti, Li, and Pt) in each patch.

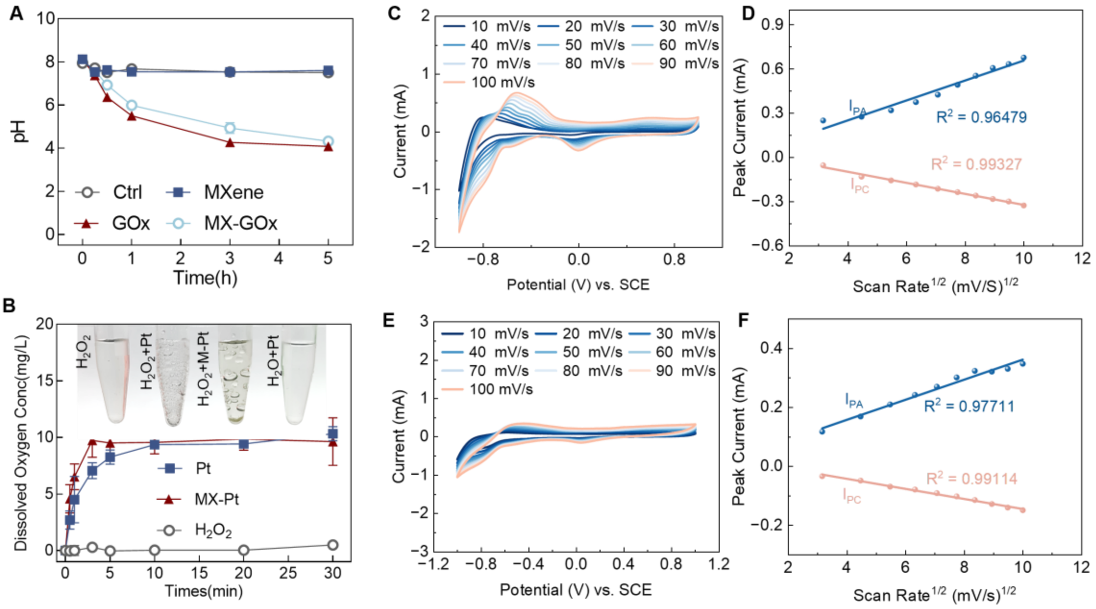

Fig. S3 Supplementary electrochemical characterization of the GEB. (**A**) Local pH variation of the MX-GOx electrode during glucose oxidation, indicating effective catalytic conversion. (**B**) Decomposition of generated H_2O_2 into O_2 by the Pt electrode, confirming its catalytic efficiency. (**C**) CV curves of the MX–Pt electrode at different scan rates. (**D**) Linear relation between peak current and the square root of scan rate for the MX–Pt electrode, indicating diffusion-controlled redox kinetics. (**E**) CV curves of the MX–GOx electrode at different scan rates. (**F**) Linear relation between peak current and the square root of scan rate for the MX–GOx electrode, indicating diffusion-controlled redox kinetics.

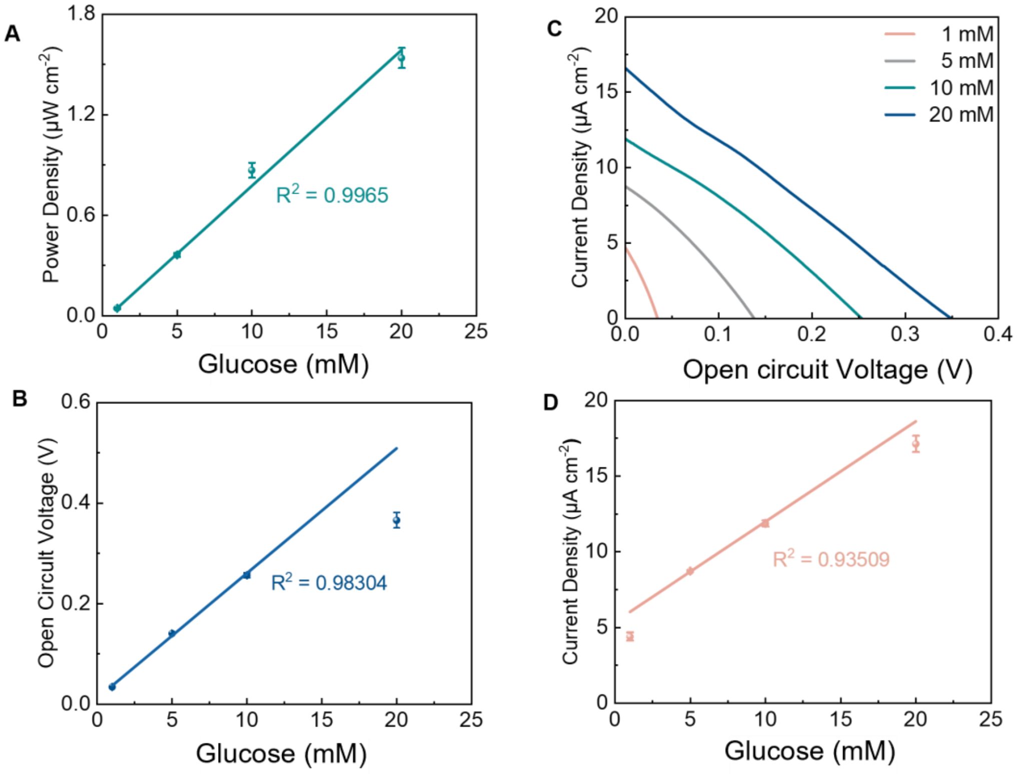

Fig. S4 performance evaluation of the GEB under varying glucose concentrations. (**A**) Power density curves of the GEB at different glucose concentrations. (**B**) Open-circuit voltage (OCV) variation from 5 to 20 mM glucose. (**C**) Current density–voltage curves of the GEB at different glucose concentrations. **(D**) Dependence of current density on glucose concentration, showing enhanced output with increasing glucose levels.

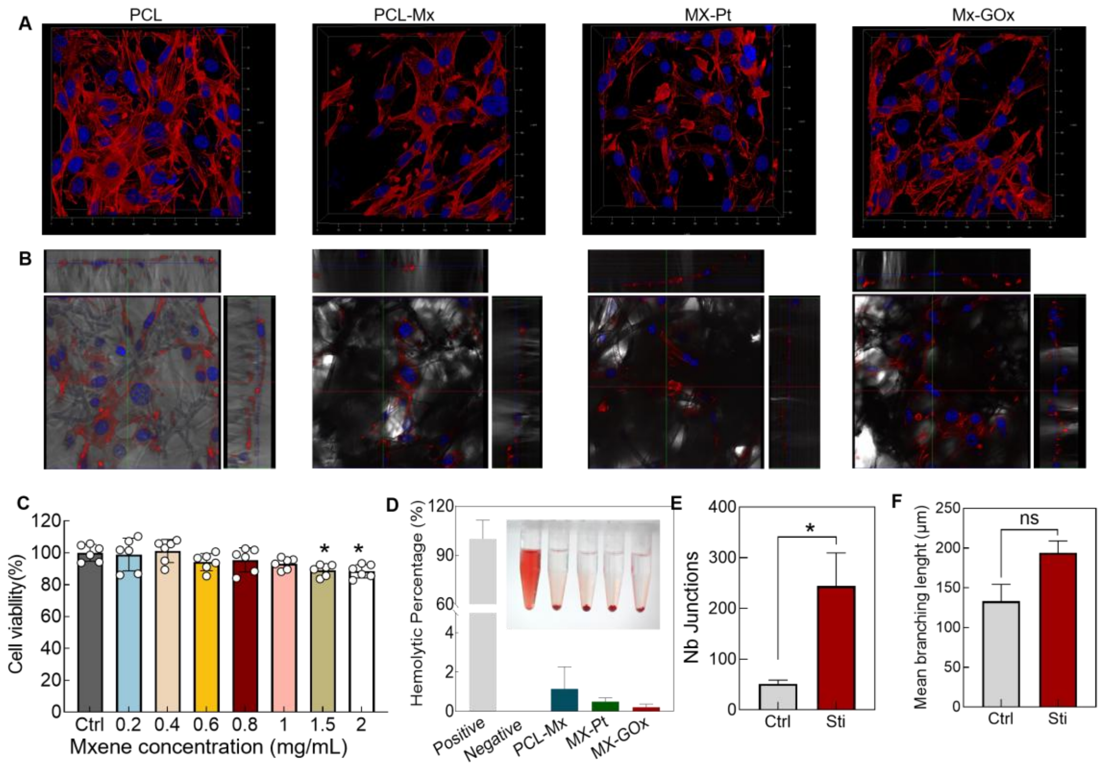

Fig. S5 biocompatibility and hemocompatibility evaluation. (**A, B**) Fluorescence images of cytoskeletal staining of HaCaT cells cultured on different materials, showing uniform adhesion and well-spread morphology on the GEB surface. (**C**) Cell viability of HaCaT cells cultured with MXene solutions at various concentrations. (**D**) Hemolysis assay results of GEB, MXene, and PCL, all exhibiting hemolysis rates below 5%, confirming good blood compatibility. (**E**) Quantitative analysis of junction number (Nb junctions) of HUVECs, showing significant enhancement on GEB. (**F**) Average branch length of HUVECs.

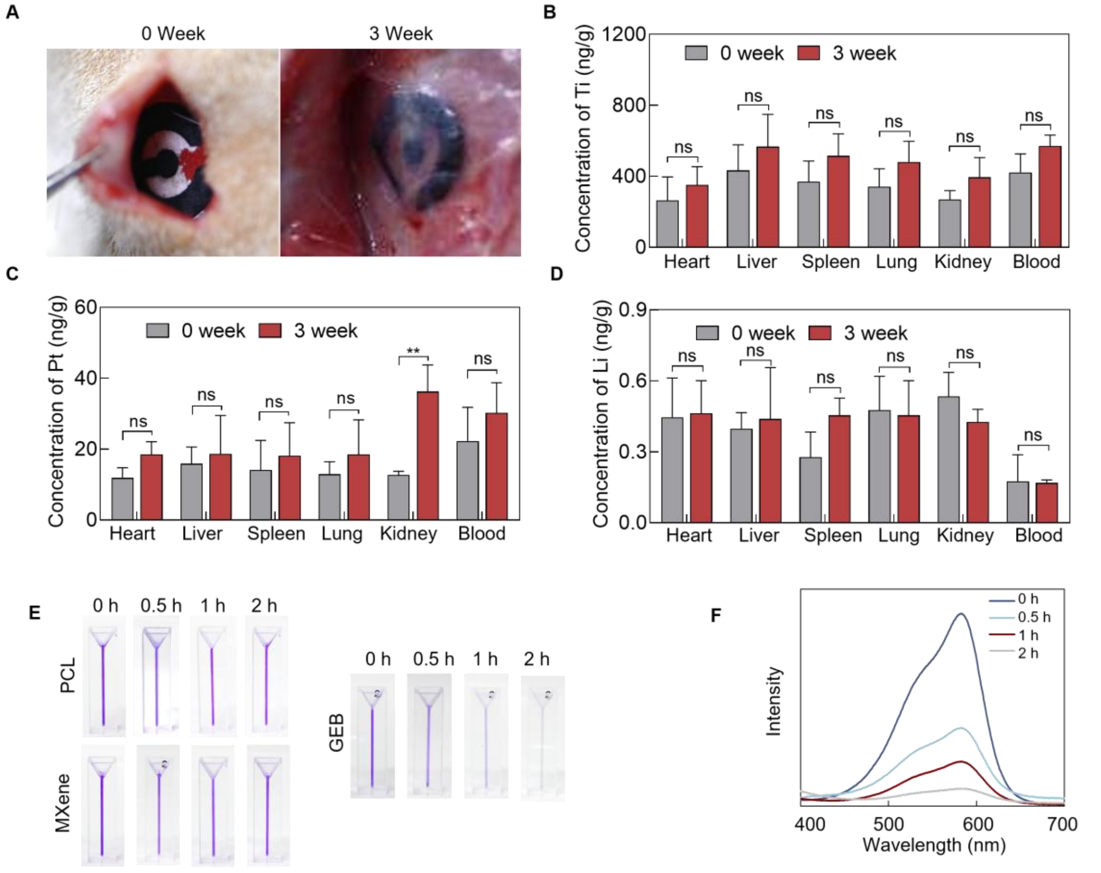

Fig. S6 In vivo elemental biodistribution of the GEB. (**A**) Representative photographs had shown the subcutaneous implantation of the GEB. (**B–D**) ICP-MS-based in vivo distribution of Ti (B), Pt (C), and Li (D) in blood and major organs at 0- and 3-weeks post-implantation (n = 3). (**E**) Photographs showing gradual decolorization of methyl violet solution after co-incubation with GEB for 2 hours, indicating ROS generation. (**F**) Time-dependent absorbance spectra of methyl violet solution confirming ROS-mediated degradation induced by GEB.

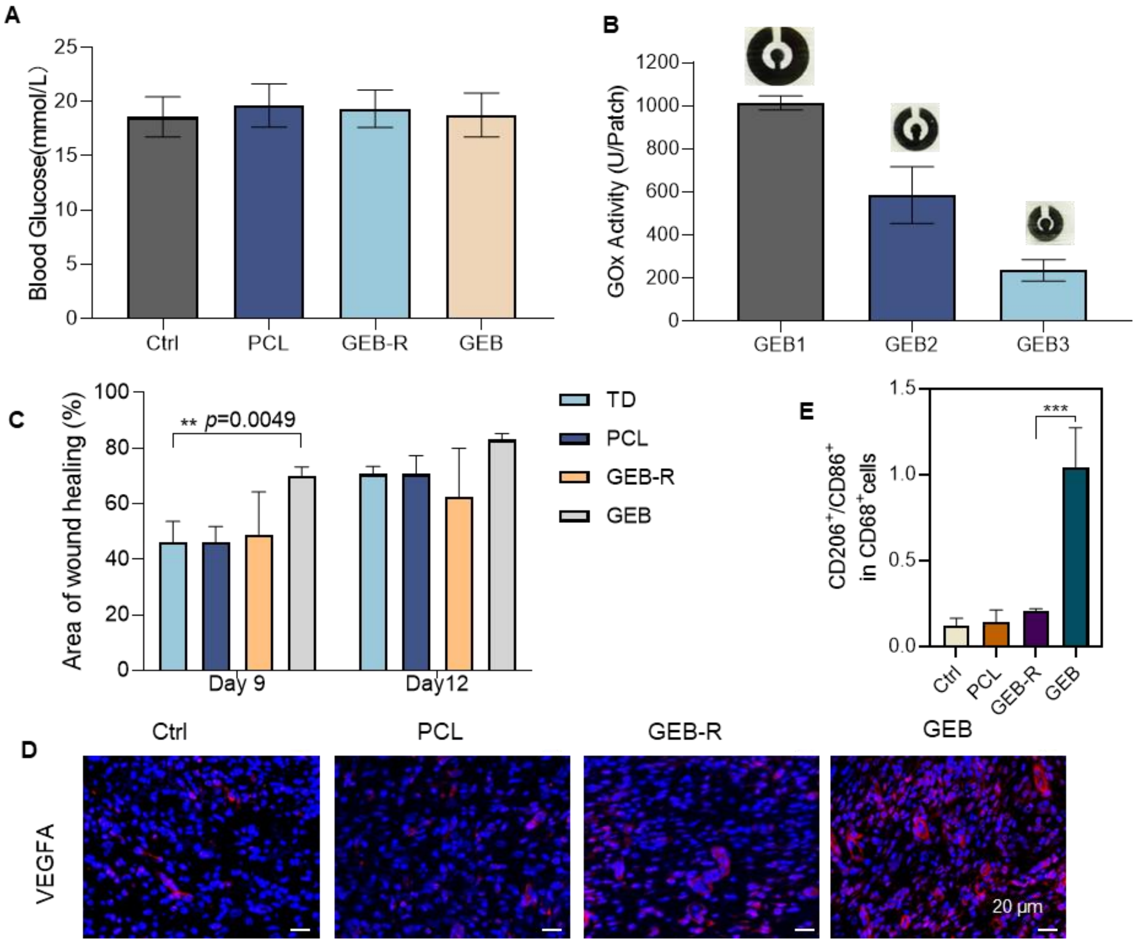

Fig. S7 Supplementary evaluation of diabetic wound model establishment and histological analysis. (**A**) Blood glucose levels of mice after streptozotocin induction (**B**) Relationship between dressing size and GOx enzyme activity, showing proportional scaling. (**C**) Quantitative analysis of wound closure rate on day 9 and day 12 for different treatment groups. (**D**) Immunofluorescence staining of VEGFA showing enhanced angiogenesis in the GEB group. (**E**) semi-quantitative analysis of CD206/CD86 expression showing increased M2 polarization in the GEB group based on immunofluorescence staining

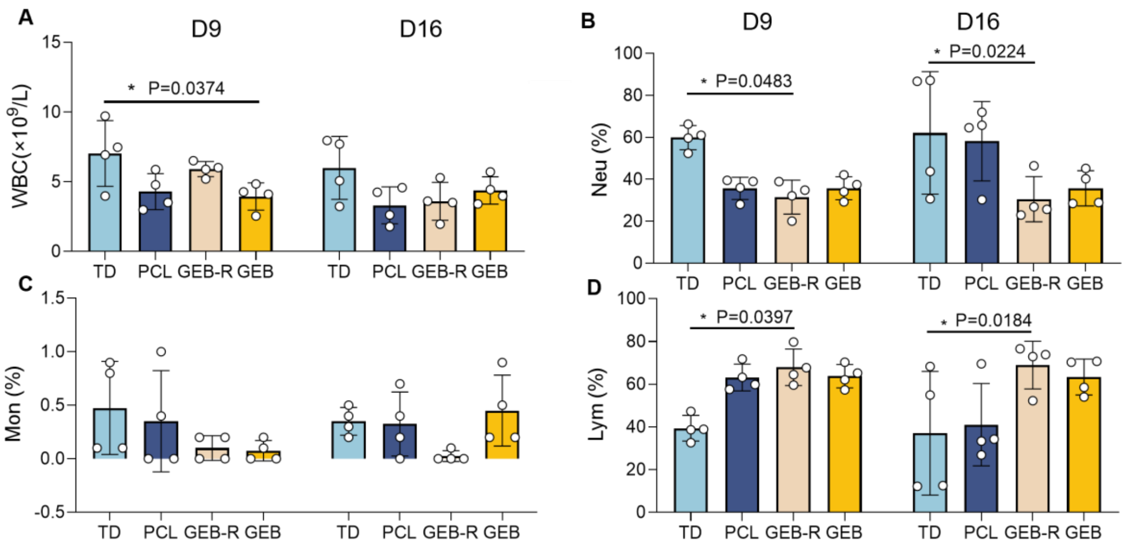

Fig. S8 Peripheral blood analysis of diabetic mice after different treatments.

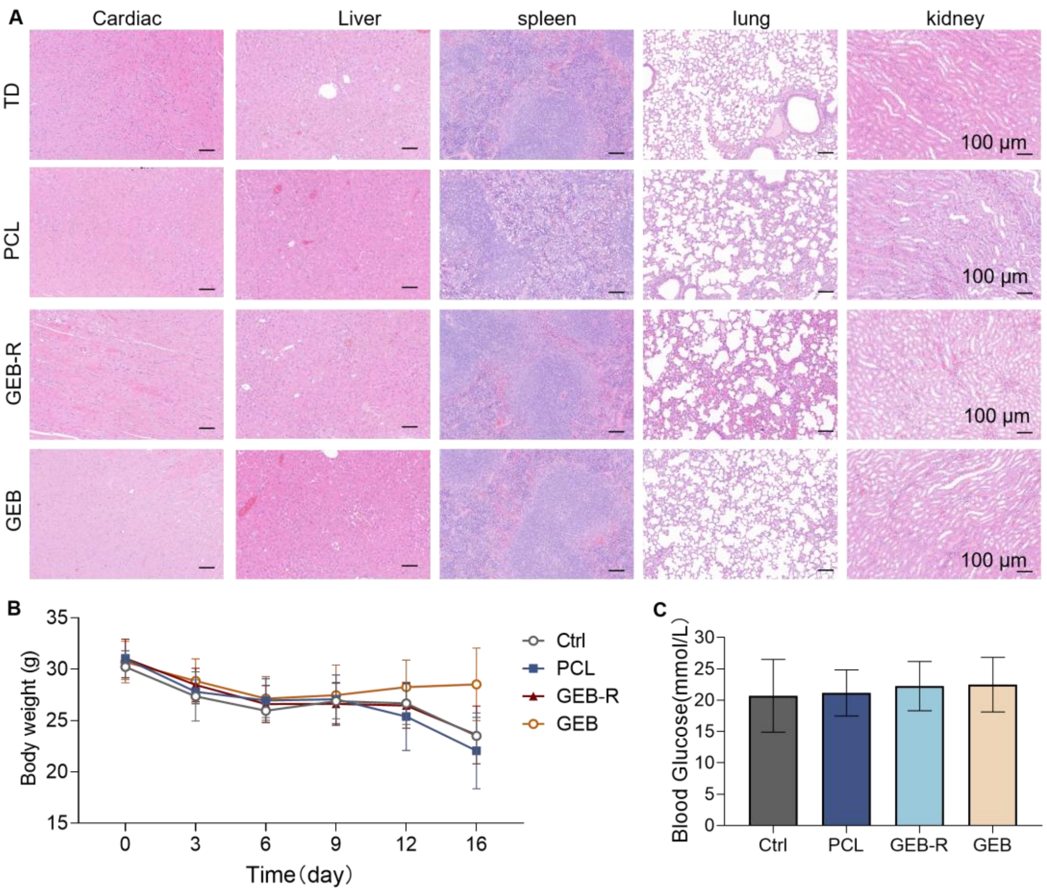

Fig. S9 Systemic biocompatibility and physiological evaluation of GEB-treated diabetic mice. (**A**) H&E staining of major organs on day 16, showing no pathological abnormalities or inflammatory lesions. (**B**) Body-weight monitoring of diabetic mice during treatment, showing attenuated weight loss in the GEB group. (**C**) Blood glucose levels on day 16, showing no significant difference among groups, confirming that wound-healing improvement is independent of glycaemic change.

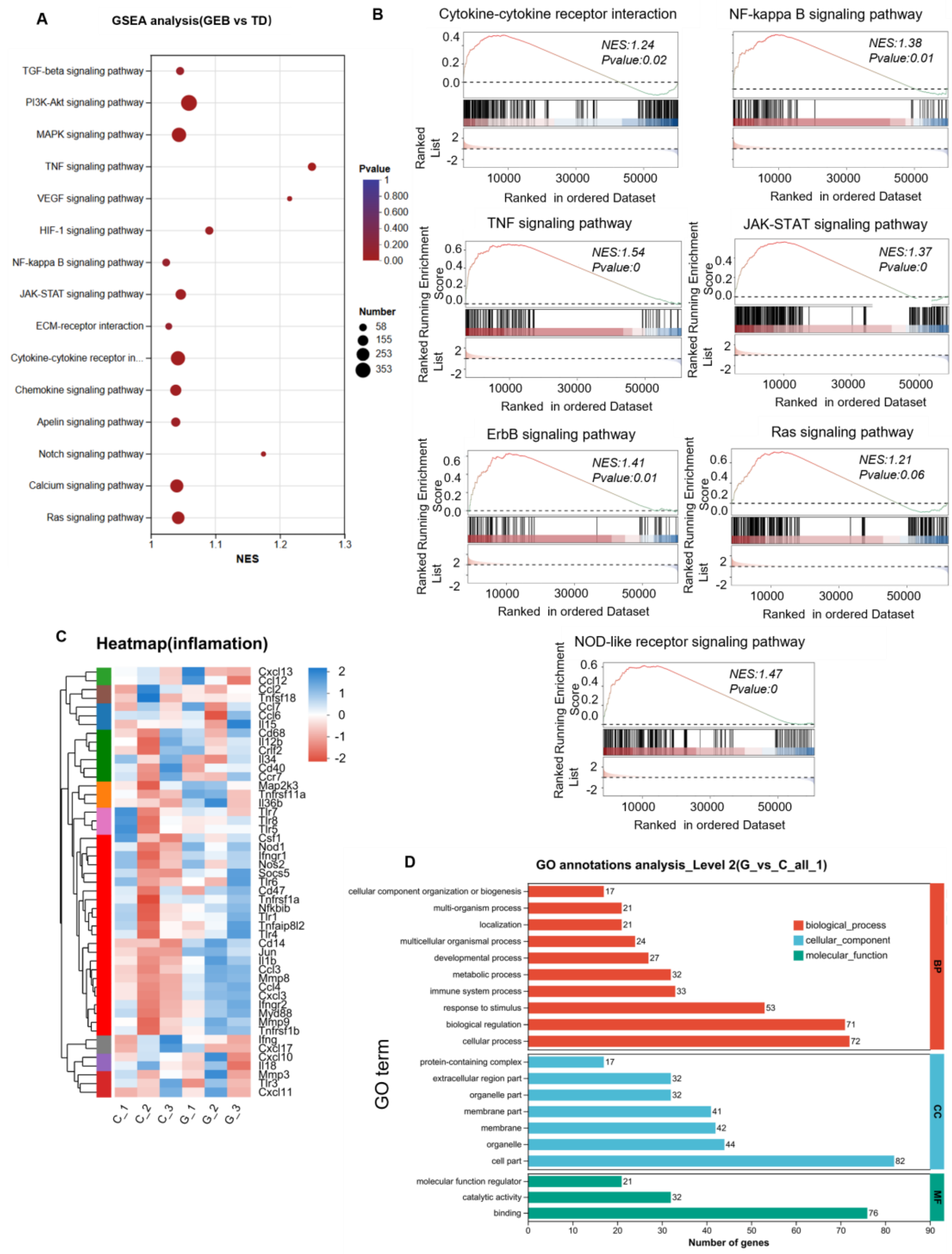

Fig. S10 Transcriptomic and functional enrichment analyses of wound tissues from diabetic mice treated with GEB. (**A**) Bubble plot of enriched immune-related pathways. (**B**) Gene Set Enrichment Analysis (GSEA) of wound tissues showing significant enrichment signaling pathways in GEB-treated mouse wounds. (**C**) Heatmap of inflammation-related genes showing downregulation of pro-inflammatory mediators in the GEB group. (**D**) Gene Ontology (GO) annotation analysis showing enrichment in cellular processes, immune regulation, metabolic activity, and developmental pathways.

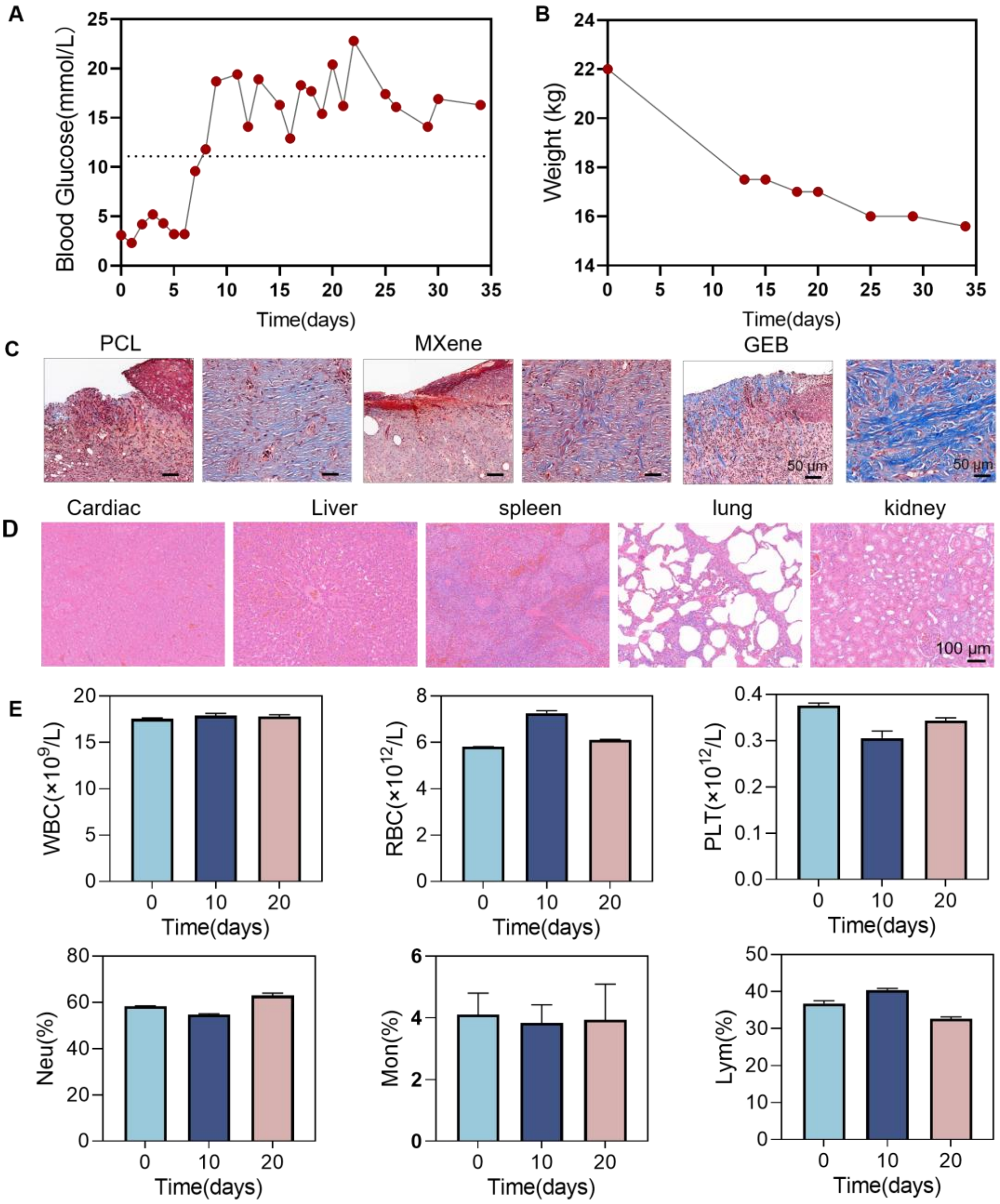

Fig. S11 Assessment of wound healing progression in diabetic porcine wound healing experiments. (**A**) Blood glucose levels in diabetic porcine showing successful induction of diabetes during the experiment. (**B**) Monitoring of body weight in diabetic pigs, showing a gradual decline within a physiologically acceptable range, consistent with typical diabetic characteristics. (**C**) Sirius Red and Masson's trichrome staining of wound tissues from different groups. (**D**) Hematoxylin-eosin (H&E) staining of major organs at the end of the study, showing no pathological abnormalities, indicating good systemic safety. (**E**) Routine blood parameter analysis confirming stable parameters throughout the treatment period in all groups, with no evidence of infection or inflammation.

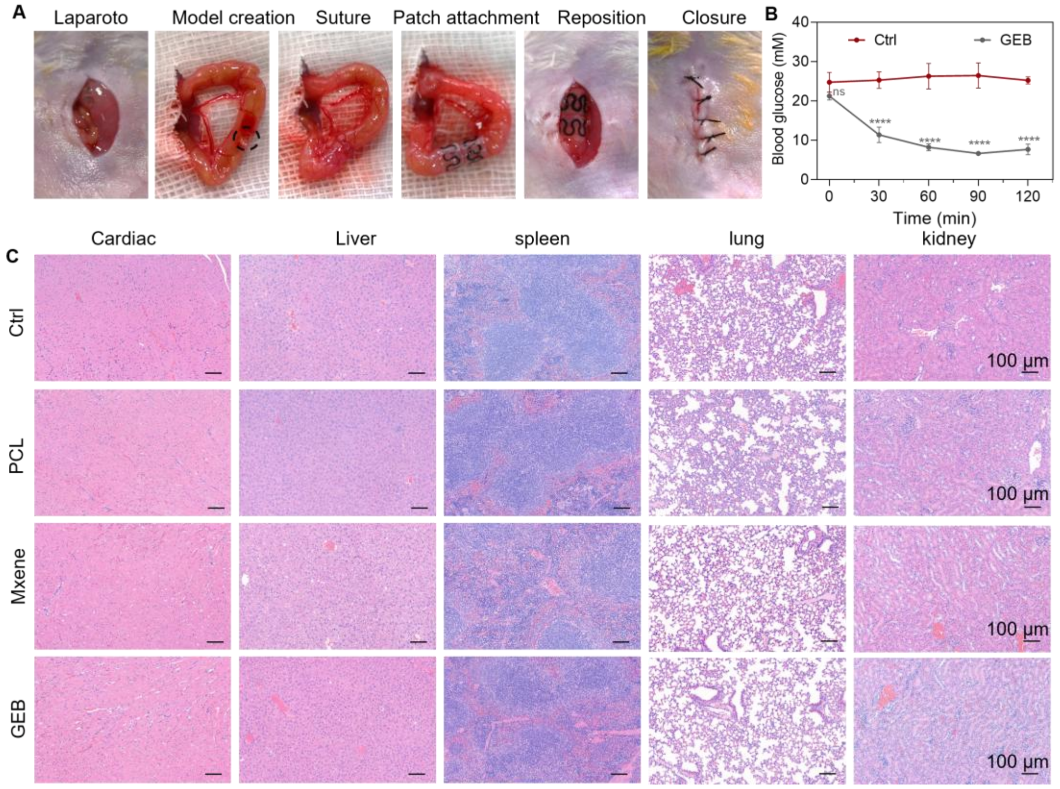

Fig. S12 Supplementary procedural and safety evaluation for intestinal wound healing experiments. (**A**) Stepwise schematic of the intestinal wound surgery, two of the images in this figure are also presented in Fig. 7B (**B**) Time-dependent changes in local glucose concentration at an intestinal wound site in hyperglycemic mice during GEB operation. (**C**) H&E staining of major organs at the end of the experiment, showing no pathological abnormalities or organ damage, confirming safety of GEB.

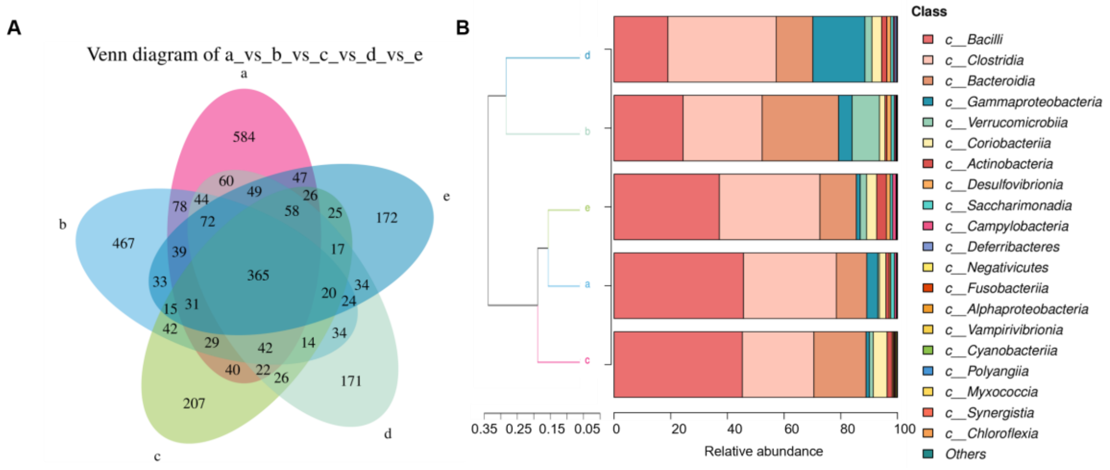

Fig. S13 Gut microbiota sequencing for intestinal wound healing experiments in diabetic mice. (**A**) Principal coordinate analysis (PCoA) of 16S rDNA sequencing data showing distinct microbial community structures between experimental groups. (**B**) Relative abundance of microbial communities at the class level, showing distinct microbial compositions among the different treatment groups, with the GEB group exhibiting the most similar microbial structure to healthy mice.

Table S1 Primer sequence used for q-PCR.

Gene name	Primer (5’-3’)	Primer (3’-5’)
Mapk1	GGTTGTTCCCAAATGCTGACT	CAACTTCAATCCTCTTGTGAGGG
Akt1	ATGAACGACGTAGCCATTGTG	TTGTAGCCAATAAAGGTGCCAT
Pik3ca	CACTCGTCACCATCAAACATGA	AGGGTTGAAAAAGCCGAAGGT
Tgfb1	CTTCAATACGTCAGACATTCGG	GTAACGCCAGGAATTGTTGCTA
EGF	GGAAGCCACGCTTACATTCAT	ACTGAGTAGAAGATCCGATCACC
Pdgfa	GCGGCGAAATTCAGTACCATC	GTCCAGACAGGTGATCGGTG
Fgf1	CAGCTCAGTGCGGAAAGTG	TGTCTGCGAGCCGTATAAAAG
Egfr	GCATCATGGGAGAGAACAACA	CTGCCATTGAACGTACCCAGA
Col1a1	TAAGGGTCCCCAATGGTGAGA	GGGTCCCTCGACTCCTACAT
β-actin	TCTGCTGGAAGGTGGACAGT	CCTCTATGCCAACACAGTGC

Table S2. Comparison of the overall performance of GEB with other reported glucose-biofuel-cell-based wound bandages.

Anode	Cathode	Substrate	Voltage (V)	Current (μA)	Implantability	Biodegrad-able	Breath-ability	Flexibility	Mass production	Refs.
MXene/GOx	MXene/Pt	PCL	0.35	-	Demonstrated	Fully	Yes	Flexibility	High	This work
Au/CuS-Graphene	Pt-Graphene	PI	0.3	-	Not applicable	No	No	Partials	Medium	(*26*)
ZIF8/GOx	ZIF8/HRP	PVA/HA, DA-PPy	-	3.5	Not demonstrated	Partials	Partials	No	Medium	(*27*)
MAF-7-SWCNT-Gox/HRP	SWCNT/AuNPs /HRP	Medical gauze	0.2	-	Not demonstrated	No	No	Partials	Medium	(*59*)
PtNi nanochains	PtNi nanocages	chitosan	0.403	-	Not demonstrated	Partials	No	Flexibility	Medium	(*60*)

REFERENCES

1. K. Khunti, Y. V. Chudasama, E. W. Gregg, M. Kamkuemah, S. Misra, J. Suls, N. S. Venkateshmurthy, J. Valabhji, Diabetes and multiple long-term conditions: A review of our current global health challenge. *Diabetes Care* **46**, 2092–2101 (2023).

2. H. Sun, P. Saeedi, S. Karuranga, M. Pinkepank, K. Ogurtsova, B. B. Duncan, C. Stein, A. Basit, J. C. N. Chan, J. C. Mbanya, M. E. Pavkov, A. Ramachandaran, S. H. Wild, S. James, W. H. Herman, P. Zhang, C. Bommer, S. Kuo, E. J. Boyko, D. J. Magliano, Idf diabetes atlas: Global, regional and country-level diabetes prevalence estimates for 2021 and projections for 2045. *Diabetes Res. Clin. Pract.* **183**, 109119 (2022).

3. B. R. Freedman, C. Hwang, S. Talbot, B. Hibler, S. Matoori, D. J. Mooney, Breakthrough treatments for accelerated wound healing. *Sci. Adv.* **9**, eade7007 (2023).

4. NCD Risk Factor Collaboration (NCD-RisC), Worldwide trends in diabetes prevalence and treatment from 1990 to 2022: A pooled analysis of 1108 population-representative studies with 141 million participants. *Lancet* **404**, 2077–2093 (2024).

5. K. A. Gallagher, J. L. Mills, D. G. Armstrong, M. S. Conte, R. S. Kirsner, S. D. Minc, J. Plutzky, K. W. Southerland, M. Tomic-Canic, Current status and principles for the treatment and prevention of diabetic foot ulcers in the cardiovascular patient population: A scientific statement from the American Heart Association. *Circulation* **149**, e232 (2024).

6. D. G. Armstrong, T. W. Tan, A. J. M. Boulton, S. A. Bus, Diabetic foot ulcers: A review. *JAMA* **330**, 62–75 (2023).

7. B. Song, M. Zhao, J. Forrester, C. McCaig, Nerve regeneration and wound healing are stimulated and directed by an endogenous electrical field in vivo. *J. Cell Sci.* **117**, 4681–4690 (2004).

8. G. Zhang, P. N. Samarawickrama, L. Gui, Y. Ma, M. Cao, H. Zhu, W. Li, H. Yang, K. Li, Y. Yang, E. Zhu, W. Li, Y. He, Revolutionizing diabetic foot ulcer care: The senotherapeutic approach. *Aging Dis.* **16**, 946–970 (2024).

9. L. Cheng, Z. Zhuang, M. Yin, Y. Lu, S. Liu, M. Zhan, L. Zhao, Z. He, F. Meng, S. Tian, L. Luo, A microenvironment-modulating dressing with proliferative degradants for the healing of diabetic wounds. *Nat. Commun.* **15**, 9786 (2024).

10. S. Matoori, A. Veves, D. J. Mooney, Advanced bandages for diabetic wound healing. *Sci. Transl. Med.***13**, eabe4839 (2021).

11. E. Kim, S. Kim, Y. W. Kwon, H. Seo, M. Kim, W. G. Chung, W. Park, H. Song, D. H. Lee, J. Lee, S. Lee, I. Jeong, K. Lim, J. U. Park, Electrical stimulation for therapeutic approach. *Interdiscip. Med.* **1**, e20230003 (2023).

12. R. Luo, J. Dai, J. Zhang, Z. Li, Accelerated skin wound healing by electrical stimulation. *Adv. Healthc. Mater.* **10**, e2100557 (2021).

13. M. Zhao, B. Song, J. Pu, T. Wada, B. Reid, G. Tai, F. Wang, A. Guo, P. Walczysko, Y. Gu, T. Sasaki, A. Suzuki, J. V. Forrester, H. R. Bourne, P. N. Devreotes, C. D. McCaig, J. M. Penninger, Electrical signals control wound healing through phosphatidylinositol-3-OH kinase-γ and PTEN. *Nature* **442**, 457–460 (2006).

14. M. R. Asadi, G. Torkaman, Bacterial inhibition by electrical stimulation. *Adv. Wound Care* **3**, 91–97 (2014).

15. P. Mohana Sundaram, K. K. Rangharajan, E. Akbari, T. J. Hadick, J. W. Song, S. Prakash, Direct current electric field regulates endothelial permeability under physiologically relevant fluid forces in a microfluidic vessel bifurcation model. *Lab Chip* **21**, 319–330 (2021).

16. L. C. Kloth, Electrical stimulation technologies for wound healing. *Adv. Wound Care* **3**, 81–90 (2014).

17. D. J. Cohen, W. J. Nelson, M. M. Maharbiz, Galvanotactic control of collective cell migration in epithelial monolayers. *Nat. Mater.* **13**, 409–417 (2014).

18. Y. Huang, K. Yao, Q. Zhang, X. Huang, Z. Chen, Y. Zhou, X. Yu, Bioelectronics for electrical stimulation: Materials, devices and biomedical applications. *Chem. Soc. Rev.* **53**, 8632–8712 (2024).

19. Y. W. Jiang, A. A. Trotsyuk, S. M. Niu, D. Henn, K. Chen, C. C. Shih, M. R. Larson, A. M. Mermin-Bunnell, S. Mittal, J. C. Lai, A. Saberi, E. Beard, S. Jing, D. L. Zhong, S. R. Steele, K. F. Sun, T. Jain, E. Zhao, C. R. Neimeth, W. G. Viana, J. Tang, D. Sivaraj, J. Padmanabhan, M. Rodrigues, D. P. Perrault, A. Chattopadhyay, Z. N. Maan, M. C. Leeolou, C. A. Bonham, S. H. Kwon, H. C. Kussie, K. S. Fischer, G. Gurusankar, K. Liang, K. L. Zhang, R. Nag, M. P. Snyder, M. Januszyk, G. C. Gurtner, Z. N. Bao, Wireless, closed-loop, smart bandage with integrated sensors and stimulators for advanced wound care and accelerated healing. *Nat. Biotechnol.* **41**, 652–662 (2023).

20. J. W. Song, H. Ryu, W. Bai, Z. Xie, A. Vazquez-Guardado, K. Nandoliya, R. Avila, G. Lee, Z. Song, J. Kim, M. K. Lee, Y. Liu, M. Kim, H. Wang, Y. Wu, H. J. Yoon, S. S. Kwak, J. Shin, K. Kwon, W. Lu, X. Chen, Y. Huang, G. A. Ameer, J. A. Rogers, Bioresorbable, wireless, and battery-free system for electrotherapy and impedance sensing at wound sites. *Sci. Adv.* **9**, eade4687 (2023).

21. E. Shirzaei Sani, C. Xu, C. Wang, Y. Song, J. Min, J. Tu, S. A. Solomon, J. Li, J. L. Banks, D. G. Armstrong, W. Gao, A stretchable wireless wearable bioelectronic system for multiplexed monitoring and combination treatment of infected chronic wounds. *Sci. Adv.* **9**, eadf7388 (2023).

22. S. R. Barman, S. W. Chan, F. C. Kao, H. Y. Ho, I. Khan, A. Pal, C. C. Huang, Z. H. Lin, A self-powered multifunctional dressing for active infection prevention and accelerated wound healing. *Sci. Adv.* **9**, eadc8758 (2023).

23. J. Xin, L. Gao, W. Zhang, X. Song, Y. Yang, W. Li, X. Zhou, H. Zhang, Z. Wang, Z. Wang, B. He, Y. Liu, T. Zhou, T. Xiong, S. Wang, S. Yuan, W. Li, S. C. J. Loo, L. Wang, L. Wei, A thermogalvanic cell dressing for smart wound monitoring and accelerated healing. *Nat. Biomed. Eng.* **10**, 80–93 (2026).

24. R. Kaveti, M. A. Jakus, H. Chen, B. Jain, D. G. Kennedy, E. A. Caso, N. Mishra, N. Sharma, B. E. Uzunoğlu, W. B. Han, T.-M. Jang, S.-W. Hwang, G. Theocharidis, B. J. Sumpio, A. Veves, S. K. Sia, A. J. Bandodkar, Water-powered, electronics-free dressings that electrically stimulate wounds for rapid wound closure. *Sci. Adv.* **10**, eado7538 (2024).

25. Y. Bai, H. Meng, Z. Li, Z. L. Wang, Degradable piezoelectric biomaterials for medical applications. *MedMat* **1**, 40–49 (2024).

26. C. Gu, L. Zhang, T. Hou, Q. Wang, F. Li, P. Gai, Laser-induced nanozyme biofuel cell-based self-powered patch for accelerating diabetic wound healing with real-time monitoring. *Adv. Funct. Mater.* **35**, 2423106 (2025).

27. X. Zhang, Z. Wang, H. Jiang, H. Zeng, N. An, B. Liu, L. Sun, Z. Fan, Self-powered enzyme-linked microneedle patch for scar-prevention healing of diabetic wounds. *Sci. Adv.* **9**, eadh1415 (2023).

28. H. Wu, Y. Wang, H. Li, Y. Hu, Y. Liu, X. Jiang, H. Sun, F. Liu, A. Xiao, T. Chang, L. Lin, K. Yang, Z. Wang, Z. Dong, Y. Li, S. Dong, S. Wang, J. Chen, Y. Liu, D. Yin, H. Zhang, M. Liu, S. Kong, Z. Yang, X. Yu, Y. Wang, Y. Fan, L. Wang, C. Yu, L. Chang, Accelerated intestinal wound healing via dual electrostimulation from a soft and biodegradable electronic bandage. *Nat. Electron.* **7**, 299–312 (2024).

29. X. F. Wang, M. L. Li, Q. Q. Fang, W. Y. Zhao, D. Lou, Y. Y. Hu, J. Chen, X. Z. Wang, W. Q. Tan, Flexible electrical stimulation device with Chitosan-Vaseline® dressing accelerates wound healing in diabetes. *Bioact. Mater.* **6**, 230–243 (2021).

30. H. Y. Xue, J. Jin, Z. Tan, K. L. Chen, G. X. Lu, Y. S. Zeng, X. L. Hu, X. C. Peng, L. M. Jiang, J. G. Wu, Flexible, biodegradable ultrasonic wireless electrotherapy device based on highly self-aligned piezoelectric biofilms. *Sci. Adv.* **10**, eadn0260 (2024).

31. Y. Shan, L. Xu, X. Cui, J. Zhang, H. Ouyang, X. Wang, J. Huang, J. Xue, K. Wang, D. Wang, E. Wang, K. Ren, D. Luo, Z. Li, A neurodevelopment-inspired self-evolving scaffold for nerve regeneration. *Cell Biomater.* **1**, 100006 (2025).

32. B. Szefler, M. Diudea, M. Putz, I. Grudzinski, Molecular dynamic studies of the complex polyethylenimine and glucose oxidase. *Int. J.Mol. Sci.* **17**,1796 (2016).

33. Z. Ma, Z. P. Cano, A. Yu, Z. Chen, G. Jiang, X. Fu, L. Yang, T. Wu, Z. Bai, J. Lu, Enhancing oxygen reduction activity of Pt-based electrocatalysts: From theoretical mechanisms to practical methods. *Angew. Chem. Int. Ed. Engl.* **59**, 18334–18348 (2020).

34. L. Sun, Q. Zhao, L. Che, M. Li, X. Leng, Y. Long, Y. Lu, Multi-stimuli-responsive weldable bilayer actuator with programmable patterns and 3D shapes. *Adv. Funct. Mater.* **34**, 2311398 (2023).

35. R. Xu, H. Xia, W. He, Z. Li, J. Zhao, B. Liu, Y. Wang, Q. Lei, Y. Kong, Y. Bai, Z. Yao, R. Yan, H. Li, R. Zhan, S. Yang, G. Luo, J. Wu, Controlled water vapor transmission rate promotes wound-healing via wound re-epithelialization and contraction enhancement. *Sci. Rep.* **6**, 24596 (2016).

36. P. Daubinger, J. Kieninger, T. Unmussig, G. A. Urban, Electrochemical characteristics of nanostructured platinum electrodes—A cyclic voltammetry study. *Phys. Chem. Chem. Phys.* **16**, 8392–8399 (2014).

37. E. Wang, M. Wu, L. Luo, X. Cui, L. Xu, R. Luo, Y. Zou, T. Le, Y. Shan, Y. Quan, Y. Bai, L. Wu, Y. Hu, S. Cheng, J. Yang, C. Zhu, D. Yu, J. Ji, Y. Ren, D. Jiang, B. Shi, H. Feng, W. Hua, Z. Li, H. Ouyang, Symbiotic biodegradable flexible supercapacitor in vivo. *Device* **3**,100724 (2025).

38. I. P. Saebo, M. Bjoras, H. Franzyk, E. Helgesen, J. A. Booth, Optimization of the hemolysis assay for the assessment of cytotoxicity. *Int. J. Mol. Sci.* **24**, 2914 (2023).

39. J. Dawi, K. Tumanyan, K. Tomas, Y. Misakyan, A. Gargaloyan, E. Gonzalez, M. Hammi, S. Tomas, V. Venketaraman, Diabetic foot ulcers: Pathophysiology, immune dysregulation, and emerging therapeutic strategies. *Biomedicines* **13**,1074 (2025).

40. X. Liu, Z. Yan, Y. Zhang, Z. Liu, Y. Sun, J. Ren, X. Qu, Two-dimensional metal–organic framework/enzyme hybrid nanocatalyst as a benign and self-activated cascade reagent for in vivo wound healing. *ACS Nano* **13**, 5222–5230 (2019).

41. P. Martin, C. Pardo-Pastor, R. G. Jenkins, J. Rosenblatt, Imperfect wound healing sets the stage for chronic diseases. *Science* **386**, eadp2974 (2024).

42. Y. Kang, L. Xu, J. Dong, X. Yuan, J. Ye, Y. Fan, B. Liu, J. Xie, X. Ji, Programmed microalgae-gel promotes chronic wound healing in diabetes. *Nat. Commun.* **15**, 1042 (2024).

43. J. Wu, H. Yuk, T. L. Sarrafian, C. F. Guo, L. G. Griffiths, C. S. Nabzdyk, X. Zhao, An off-the-shelf bioadhesive patch for sutureless repair of gastrointestinal defects. *Sci. Transl. Med.* **14**, eabh2857 (2022).

44. N. Vatankhah, Y. Jahangiri, G. J. Landry, R. B. McLafferty, N. J. Alkayed, G. L. Moneta, A. F. Azarbal, Predictive value of neutrophil-to-lymphocyte ratio in diabetic wound healing. *J. Vasc. Surg.* **65**, 478–483 (2017).

45. S. H. Shin, S. K. Han, S. H. Jeong, W. K. Kim, Potential of oncostatin M to accelerate diabetic wound healing. *Int. Wound J.* **11**, 398–403 (2014).

46. R. A. Crompton, H. Williams, L. Campbell, L. Hui Kheng, C. Saville, D. M. Ansell, A. Reid, J. Wong, L. A. Vardy, M. J. Hardman, S. M. Cruickshank, An epidermal-specific role for arginase1 during cutaneous wound repair. *J. Invest. Dermatol.* **142**, 1206–1216.e8 (2022).

47. R. Wang, S. Gu, Y. H. Kim, A. Lee, H. Lin, D. Jiang, Diabetic wound repair: From mechanism to therapeutic opportunities. *MedComm* **6**, e70406 (2025).

48. D. A. Rappolee, D. Mark, M. J. Banda, Z. Werb, Wound macrophages express TGF-α and other growth factors in vivo: Analysis by mRNA phenotyping. *Science* **241**, 708–712 (1988).

49. N. Yadu, M. Singh, D. Singh, S. Keshavkant, Mechanistic insights of diabetic wound: Healing process, associated pathways and microRNA-based delivery systems. *Int. J. Pharm.* **670**, 125117 (2025).

50. X. Zhang, B. Gong, F. Rao, H. Hu, F. Tian, Y. Lu, L. Zhang, Y. Xia, J. Xue, Spatiotemporally controlled delivery of biological effectors from nanofiber scaffolds accelerates skin wound healing in porcine models. *Sci. Adv.* **11**, eadz5302 (2025).

51. C. J. Chiu, A. H. McArdle, R. Brown, H. J. Scott, F. N. Gurd, Intestinal mucosal lesion in low-flow states. I. A morphological, hemodynamic, and metabolic reappraisal. *Arch. Surg.* **101**, 478–483 (1970).

52. V. Singh, G. Lee, H. Son, H. Koh, E. S. Kim, T. Unno, J. H. Shin, Butyrate producers, "The Sentinel of Gut": Their intestinal significance with and beyond butyrate, and prospective use as microbial therapeutics. *Front. Microbiol.* **13**, 1103836 (2022).

53. E. Tören, A. A. Mazari, Pullulan/collagen scaffolds promote chronic wound healing via mesenchymal stem cells. *Micro* **4**, 599–620 (2024).

54. J. P. Perdew, K. Burke, M. Ernzerhof, Generalized gradient approximation made simple. *Phys. Rev. Lett.* **77**, 3865–3868 (1996).

55. G. Kresse, D. Joubert, From ultrasoft pseudopotentials to the projector augmented-wave method. *Phys. Rev. B* **59**, 1758–1775 (1999).

56. L. Xu, E. Wang, Y. Kang, D. Fu, L. Luo, Y. Quan, Y. Xi, J. Huang, X. Cui, J. Zeng, D. Jiang, B. Shi, H. Feng, H. Ouyang, C. Chen, Z. Li, Schottky nanodiodes array enabled triboelectric nanosecond pulse generator for ultralow-cost tumor therapy. *Device* **3**, 100721 (2025).

57. H. Ouyang, D. Jiang, Y. Hu, S. Cheng, Z. Zhang, B. Shi, E. Wang, J. Xue, Y. Shan, L. Xu, Y. Zou, S. Weng, H. Li, H. Niu, M. Gu, L. Luo, S. Chao, P. Tan, Y. Yao, N. Wang, Y. Fan, Z. L. Wang, W. Hua, Z. Li, Symbiotic transcatheter pacemaker for lifelong energy regeneration and therapeutic function in porcine disease model. *Nat. Biomed. Eng.* **2026**, 10.1038/s41551-025-01604-4 (2026).

58. E. Wang, L. Xu, L. Luo, Y. Ren, Y. Quan, T. Le, J. Xue, C. Zhu, J. Huang, X. Cui, D. Jiang, B. Shi, H. Feng, J. Zhang, Z. Li, H. Ouyang, Tissue-fluid-driven symbiotic electronic textile accelerates and guides tissue self-healing. *Cell Biomater.* **2**, 100375 (2026).

59. L. Wang, Q. Su, Y. Liu, T. Yimamumaimaiti, D. Hu, J. J. Zhu, J. R. Zhang, A self-powered and drug-free diabetic wound healing patch breaking hyperglycemia and low H_2O_2 limitations and precisely sterilizing driven by electricity. *Chem. Sci.* **13**, 12136–12143 (2022).

60. Z. Lin, Y. Wu, Y. Wang, P. Su, X. Li, Y. Zou, K. Chen, Y. Li, J. Zhou, T. Ye, Y. Qi, W. Wang, Flexible patterned fuel cell patches stimulate nerve and myocardium restoration. *Adv. Mater.* **37**, e2416410 (2025).
